# Tunable and computationally efficient framework for retrofitting: Budget allocation using income and spatial equity

**DOI:** 10.1093/pnasnexus/pgag162

**Published:** 2026-05-12

**Authors:** Aparna Kishore, Kaarthik Sundar, Deepjyoti Deka, Madhav V Marathe

**Affiliations:** Department of Computer Science, University of Virginia, Charlottesville, VA 22904, USA; Biocomplexity Institute, University of Virginia, Charlottesville, VA 22903, USA; Computational Intelligence & Modeling Group, Los Alamos National Laboratory, Los Alamos, NM 87545, USA; MIT Energy Initiative, Massachusetts Institute of Technology, Cambridge, MA 02142, USA; Department of Computer Science, University of Virginia, Charlottesville, VA 22904, USA; Biocomplexity Institute, University of Virginia, Charlottesville, VA 22903, USA

**Keywords:** retrofitting, optimization, budget allocation, IW_ϵ_ index

## Abstract

The residential sector accounts for roughly 35% of US electricity use. Retrofitting residential buildings has been proposed as an effective strategy to reduce energy costs and improve individuals’ quality of life and health. Federal and state funding plays a crucial role in supporting these initiatives by providing financial assistance through grants, tax credits, and low-interest loans. Key challenges here include selecting which households to serve and determining assistance levels so that savings are not skewed toward particular regions or income brackets. We propose RAISE (Retrofitting Allocation using Income & Spatial Equity), a framework that allocates a state’s retrofit budget to maximize total energy savings while ensuring fair distribution across counties and income groups. RAISE estimates prospective savings through hourly household consumption models—pre- and post-retrofit—that integrate demographics, weather data, solar irradiance, appliance inventories, and survey inputs. At the core of RAISE’s budget allocation is an income-weighted ϵ (${\text{IW}}_{\epsilon }$) index, where parameter ϵ∈[0,1] tunes the balance between efficiency and equity by enforcing two tiers of constraints: intercounty equity weighted by each county’s median income, and intracounty equity among households based on individual incomes. By adjusting ϵ, policymakers can trade off equity against total savings to match policy goals. A Virginia case study shows that RAISE—across varying ϵ settings—delivers 60–101% greater energy savings than current approaches while markedly reducing the number of low-income households receiving <10% of their retrofit cost. This two-tiered method is both computationally efficient and offers precise control over geographic and income-based allocation disparities.

Significance statement Aging buildings increase energy use and utility costs, making residential retrofitting essential for reducing emissions. However, allocating limited retrofit budgets efficiently and equitably remains difficult. We introduce RAISE (Retrofitting Allocation using Income & Spatial Equity), a tunable framework that distributes funds across both counties and income groups to balance energy savings and equity. By integrating household demographics and real-world datasets with an income-weighted ${\text{IW}}_{\epsilon }$ index, we show that RAISE achieves higher energy savings than baseline policies while reducing the number of low-income households receiving inadequate support. By incorporating an income-weighted index, the framework allows policymakers to adjust equity priorities, revealing trade-offs between efficiency and distributional outcomes.

## Introduction

Retrofitting has received global attention because of energy savings, increased comfort, and lower utility bills. International initiatives like the European Union’s Renovation Wave Initiative (1) and Canada’s Greener Homes Initiative (2) reflect an increasing focus on large-scale renovations. Approximately 60% of US homes were built before 1980 (3), making energy efficiency upgrades essential. Older buildings consume more energy, leading to higher costs and environmental impact. Retrofitting addresses these issues by improving quality of life, reducing emissions, and lowering energy expenses. This is particularly true for households in the state of Virginia (VA), a major global hub for data centers, that are old (4) and face increasing energy costs due to greater grid strain.

In the United States, a $279 billion retrofitting investment across residential, commercial, and institutional sectors could generate over $1 trillion in energy savings over 10 years—about 30% of the nation’s annual electricity costs (5). To support these efforts, various federal and state programs provide financial assistance for retrofitting, for instance, the Weatherization Assistance Program (WAP) (6), the Low Income Home Energy Assistance Program (LIHEAP), the residential clean energy credit (7), and the energy-efficient home improvement credit (8). These programs are aimed at multiple disparate outcomes, like assisting low-income households with their energy-saving home improvement and retrofitting costs and providing financial incentives for renewable energy installations. Together, they have demonstrated energy savings of 20–35% for low-income households through retrofitting measures (9). Recent research on energy justice in the US highlights that disparities in energy efficiency, retrofitting, and household energy burden are closely related to factors such as race, income, and the age of housing (10–12). Studies conducted by Reames and Hernández emphasize that structural inequities in the US housing stock and energy systems disproportionately impact low-income and minority households. This underscores the urgent need for equitable retrofitting policies (13, 14).

Implementing retrofitting measures on a state-wide scale presents several challenges, including selecting households and determining the level of financial assistance to provide. Without careful selection, allocations may not appropriately support households with high energy burden (15, 16). Such questions on equitable decision-making have also been raised in alternate areas in the energy sector like cost-sharing in retrofitting (17), grid topology optimization (18), resource coordination (19), grid planning (20), and grid control design (21). Spatial disparities in infrastructure access, an inherent characteristic of urbanization, have also been examined in broader contexts (22). For residential retrofitting, past studies examine various factors, such as energy efficiency (23), cost optimization (24, 25), improvements in air quality and comfort (26, 27), and income-based strategies for assisting low-income households (28, 29). As a strategy to maximize energy savings alone may not be socially conscious, most current retrofitting policies prioritize an income-based allocation to upgrade low-income households. While this approach is socially motivated, it could yield greater energy savings by careful selection of the energy-burdened households. Data-driven strategies have been leveraged for performance metrics, energy audits, and machine learning techniques to determine optimal retrofitting strategies for buildings (30). Researchers have balanced such retrofitting models inside various budget allocation strategies (31, 32). Although these strategies show promise, they often limit policymakers’ ability to control and adjust the allocation process to effectively balance between the dual goals of serving low-income households and improving total energy savings. Our framework is designed to provide transparent quantification and to help achieve two key goals: prioritizing overall energy savings and ensuring that household selection is based on income. This approach is particularly crucial for energy-stressed states like VA, which have seen rapid growth in energy-intensive infrastructure. A comprehensive literature review of efforts in retrofitting budget allocation is provided in the Supplementary information.

**Figure 1:**
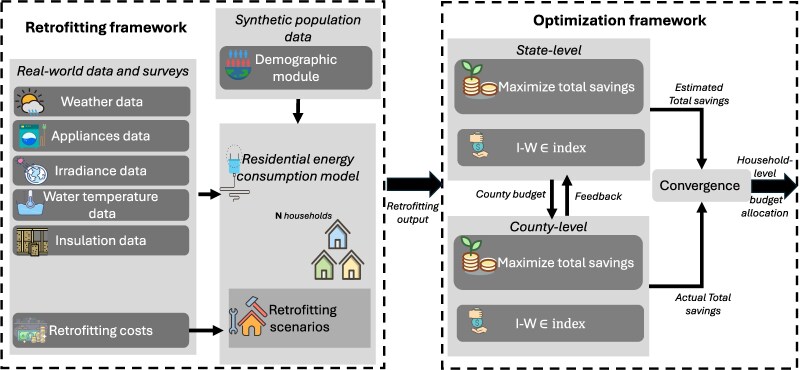
Block diagram for RAISE framework for retrofitting budget allocation. The first block outlines a retrofitting framework that incorporates detailed demographic data and real-world surveys and reports to estimate residential energy demand before and after retrofitting. The second block includes a two-tier optimization framework that allocates state budget to maximize estimated savings and ensuring income-weighted equitable distribution between and within counties of the state. The equitable distribution of budget is managed by using a tunable ${\text{IW}}_{\epsilon }$ index, at each tier.

To address this challenge, we propose a two-part framework for allocating a state’s retrofitting budget, termed RAISE (Retrofitting Allocation using Income & Spatial Equity), as illustrated in Fig. 1. RAISE includes a *retrofitting framework* that considers a population characterized by geographic and demographic attributes and incorporates real-world surveys and datasets on the energy impacts of key factors such as weather conditions, appliance usage, solar irradiance, temperature, and insulation costs. The retrofitting framework uses this information to generate hourly household energy demand under both pre- and postretrofitting scenarios (32). The generated data are then fed into an *optimization framework* designed to allocate retrofitting budgets to maximize energy savings under income-aware constraints. Specifically, our approach involves budget allocations to be constrained via a tunable income-weighted index, referred to as the ${\text{IW}}_{\epsilon }$ index. Here, ϵ∈[0,1] is a tunable parameter that enhances an equitable distribution of funds across recipients.^a^.

In RAISE, equity is defined as an income-weighted allocation of resources that prioritizes reducing disparities across households and regions, rather than equal allocation. This definition is operationalized through the income-weighted ${\text{IW}}_{\epsilon }$ index. A budget allocation is considered fair if it satisfies a user-defined threshold ϵ with respect to this index, ensuring that no household or county receives a disproportionately large share of the total relative to its income. In this formulation, fairness is the mathematical property enforced by the constraint, whereas equity is the policy objective that guides the allocation. Importantly, RAISE does not prescribe a single notion of equity. Instead, it provides a tunable framework in which the parameter ϵ enables navigation of trade-offs between efficiency (energy savings) and distributional goals. This corresponds to selecting a point along a Pareto frontier, where higher equity requirements may limit achievable energy savings, and vice versa. In the broader literature on social choice and resource allocation, equity is recognized as an inherently normative and multicriteria concept for which no single universally accepted definition exists (33–36). Arrow’s Impossibility Theorem (34) shows that in general, no allocation rule can simultaneously satisfy all seemingly reasonable fairness criteria for every stakeholder. In this context, any operational definition of equity necessarily reflects a particular set of priorities and trade-offs. RAISE therefore does not prescribe a single notion of equity but instead provides a transparent framework in which the distributional assumptions are made explicit and the parameter ϵ allows policymakers to navigate trade-offs between efficiency and equity according to their own priorities.

The optimization inside RAISE occurs in two iterative steps. First, the state-level budget is distributed *among counties* to maximize their cumulative energy savings while ensuring an equitable allocation, parameterized through the ${\text{IW}}_{\epsilon }$ index, where each county’s weight is given by its median household income. Second, the county-level budget is allocated *among its households* to maximize total energy savings while limiting the income-weighted disparities, again through the ${\text{IW}}_{\epsilon }$ index. Feedback from the county-level solution is sent back to the state-level problem to refine the county-wide budget allocations. These two steps are iterated until the estimated total savings at the two levels converge, as discussed in detail in Methods section.

This two-level allocation approach reflects what we term *spatial equity* in RAISE—the allocation of resources across geographic units based on their aggregate characteristics. The structure is consistent with major US retrofit and energy assistance programs, including the Community Development Block Grant Program (CDBG) (37) and the WAP, which allocate funding first across regions using area-level indicators such as low-income population shares and climate conditions, and only subsequently to individual households. A key advantage of RAISE is that while maximizing energy savings, it guarantees by design, that the budget distribution—both across and within counties—adheres to the user-defined income-weighted equitable standard, parameterized by the value of ϵ in the ${\text{IW}}_{\epsilon }$ index.

Our contributions are as follows: First, we present a novel framework—RAISE—that integrates retrofitting and optimization models for equitable budget allocation to maximize total energy savings from retrofitting. Second, RAISE includes ${\text{IW}}_{\epsilon }$ index, a metric that regulates the budget distribution through a constraint in the allocation problem. This index enables a two-tiered equitable allocation: (i) distributing funds among counties based on median household income and (ii) allocating funds within counties based on individual household income. Third, we apply RAISE to the US state of VA and perform income-based targeted experiments to analyze the impact of the ${\text{IW}}_{\epsilon }$ index on total budget and savings distribution among population groups and counties. RAISE shows significant gains in energy savings between 60 and 101% while providing at par or enhanced budget allocation to very low-income families when compared with current budget allocation strategies. Four, we evaluate RAISE across different income-based population groups, including low and moderate income (LMI), very low and low income (V+LI), and very low income (VLI), to highlight its flexibility. The framework is scalable, modular, and adaptable, enabling it to function effectively across any targeted demographic. This makes it especially valuable for ensuring equitable and efficient budget allocation across targeted households. While RAISE in its presented form enforces income-based equitable budget allocation, the ${\text{IW}}_{\epsilon }$ index based framework is generalizable and can include other sociodemographic metrics of equity within the allocation problem.

## Main results

We examine RAISE’s output in allocating 1 billion dollars to retrofit households (3 million) for the entire state of VA.^b^ The households were filtered based on two criteria: (i) the household annual income does not exceed $200,000 and (ii) the selected households must have an average daily saving (Wh) to retrofitting cost ($) ratio of at least 0.002 Wh/$. The threshold of 0.02 Wh/$ per day is taken as it aligns with state and federal programs, such as the WAP and the LIHEAP, that mandate retrofitting funding only to households with lifetime monetary savings-to-investment ratio (SIR) >1. The relationship with SIR is detailed in Supplementary information under selection criteria. These households in VA belong to one of the following nonoverlapping income categories:

- VLI—785,041 households
- Low-income (LI)—833,697 households
- Medium-income (MI)—452,463 households, and,
- High-income (HI)—1,066,424 households.

More details of the population, income groups, selection criteria, and related statistics in VA are presented in the Supplementary information. As a *baseline*, we consider a realistic allocation policy, similar to current state programs, that prioritizes very low and low-income households that have a ratio of average daily savings (Wh) to retrofitting costs of at least 0.002 Wh/$. To demonstrate RAISE’s flexibility, we consider three settings for ${\text{IW}}_{\epsilon }$ index: *low* (ϵ=0.5), *medium* (ϵ=0.7), and *high* (ϵ=0.9), that progressively increases the focus on reducing income-weighted disparity in allocated budget. In addition, we performed a sensitivity analysis considering various energy consumption scenarios, including maximum energy savings to address extreme weather events or peak load conditions, rather than relying solely on average savings. The results indicate that the trends are consistent across the three ${\text{IW}}_{\epsilon }$ index values. A more detailed analysis using various energy-saving settings can be found in the Supplementary information under Sensitivity analysis.

Our analysis shows that the proposed RAISE framework offers a computationally efficient and tunable allocation policy that improves upon the baseline in the following key findings:

- Increases in the aggregated energy savings by 60–101% compared to the baseline approach.
- Decreases the percentage of households with very low allocated budget (measured relative to the desired retrofitting cost) by 8%. This makes the allocation more equitable for households across income levels.
- Flexibility, where increasing the value of ϵ in the ${\text{IW}}_{\epsilon }$ index decreases the total savings but improves the disparity between income groups (within and across counties).

We discuss each of these findings in greater detail next. We refer the reader to Income classification and Selection criteria under Supplementary information for a comprehensive description of input settings. We also provide a detailed analysis of energy consumption and the budget allocated to different income categories under various ${\text{IW}}_{\epsilon }$ index settings in Supplementary information under Energy savings and budget allocation. The results show that as the ${\text{IW}}_{\epsilon }$ index increases, the proportion of the budget allocated to the VLI group rises substantially, while the shares for high and medium-income categories diminish. Additional analysis on the 1 billion dollar investments is presented in Supplementary information. We also study retrofitting allocation under larger budgets up to 5 billion dollars and present results for it in Supplementary information.

### Aggregated energy savings

**Figure 2:**
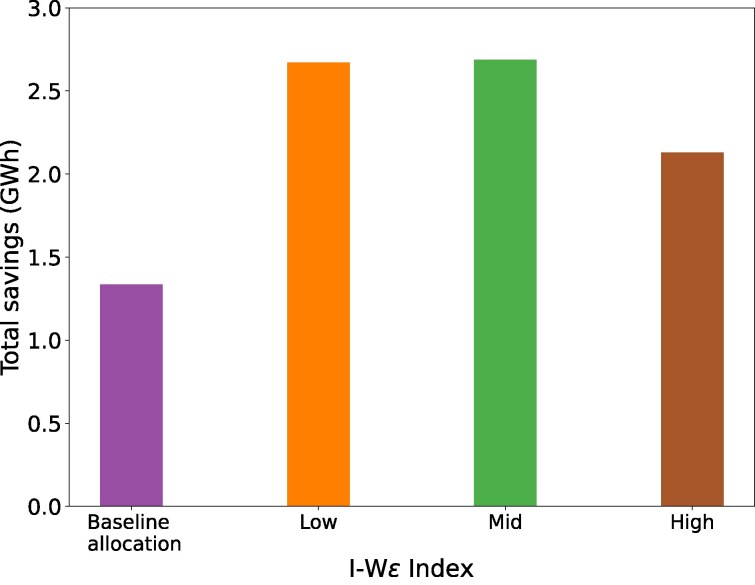
Total savings (GWh) comparison for various ${\text{IW}}_{\epsilon }$ index in RAISE: The *x*-axis represents the different levels of ${\text{IW}}_{\epsilon }$ index, and the *y*-axis represents the total savings in the state of VA in GWh. The first bar is a baseline strategy to maximize budget allocation to very low and low-income population. The three ${\text{IW}}_{\epsilon }$ Indices are: *low* (ϵ=0.5), *medium* (ϵ=0.7), and *high* (ϵ=0.9), respectively.

We compare the aggregated energy savings for different budget allocation approaches in Fig. 2. The first purple bar is a *baseline* income-based allocation that selects low-income filtered households based on the decreasing order of their income. The remaining three bars, orange, green, and brown, represent our two-level approach using different values of ϵ (low, medium, and high) for the ${\text{IW}}_{\epsilon }$ index. The figure indicates that all ${\text{IW}}_{\epsilon }$ Indices result in an energy savings improvement of at least 60.6% compared to the baseline. As expected, the savings are greater by 101% for low and medium ϵ than for high ${\text{IW}}_{\epsilon }$ index. However, this decrease in savings will lead to an improvement in the distribution of budget among very low-income households across the state, as demonstrated next.

Observation 1 RAISE results in an increase in savings by 101% in low and medium ${\text{IW}}_{\epsilon }$ index settings and an increase in savings of 60% with better budget distribution among low-income households at high ${\text{IW}}_{\epsilon }$ index settings.

### Retrofitting coverage ratio

At both county level and household level, we assess the distribution of the allocated budget relative to the retrofitting cost, which we term as retrofitting coverage ratio. This ratio indicates the what proportion of the budget desired by a household or county is allocated.

#### County-level insights

**Figure 3:**
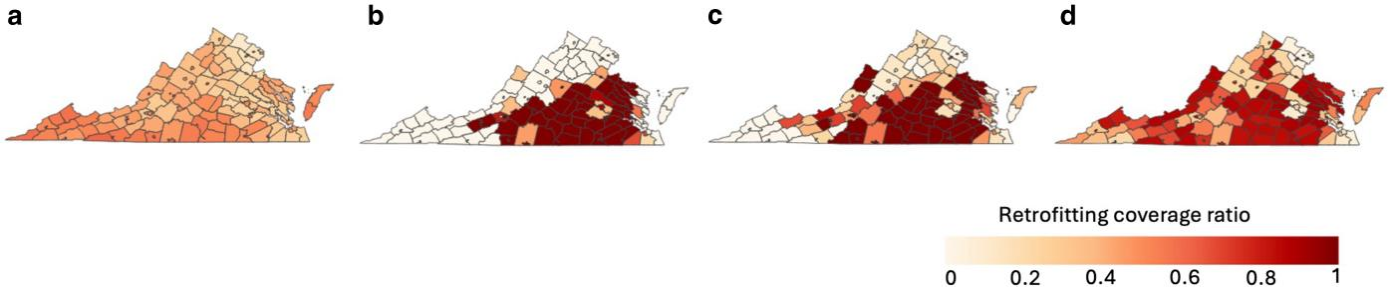
Retrofitting coverage ratio in counties, for different ${\text{IW}}_{\epsilon }$ index in RAISE: a) Baseline: to maximize budget allocation to very low and low-income population. b) Low ${\text{IW}}_{\epsilon }$ index with objective of maximizing savings. c) Medium ${\text{IW}}_{\epsilon }$ index with the objective of maximizing savings. d) High ${\text{IW}}_{\epsilon }$ index with the objective of maximizing savings. As the ${\text{IW}}_{\epsilon }$ index increases, the geographical spread for retrofitting coverage also increases.

We visualize the retrofitting coverage ratio using a VA choropleth map as shown in Fig. 3. Figure 3a illustrates that the baseline allocation results in a state-wide low retrofitting coverage ratio. With RAISE, a low ${\text{IW}}_{\epsilon }$ index provides significantly high retrofitting coverage to counties in the central-eastern and piedmont regions but negligible ratios in other regions. As the ${\text{IW}}_{\epsilon }$ index increases to medium levels, funding begins to be distributed to the northern and western counties while slightly reducing the budget allocated to certain counties that produce comparatively lower savings. At a high ${\text{IW}}_{\epsilon }$ index, RAISE’s budget distribution becomes even more widespread, allowing additional counties to receive funds for retrofitting, although this comes at the cost of lower total aggregate savings.

**Figure 4:**
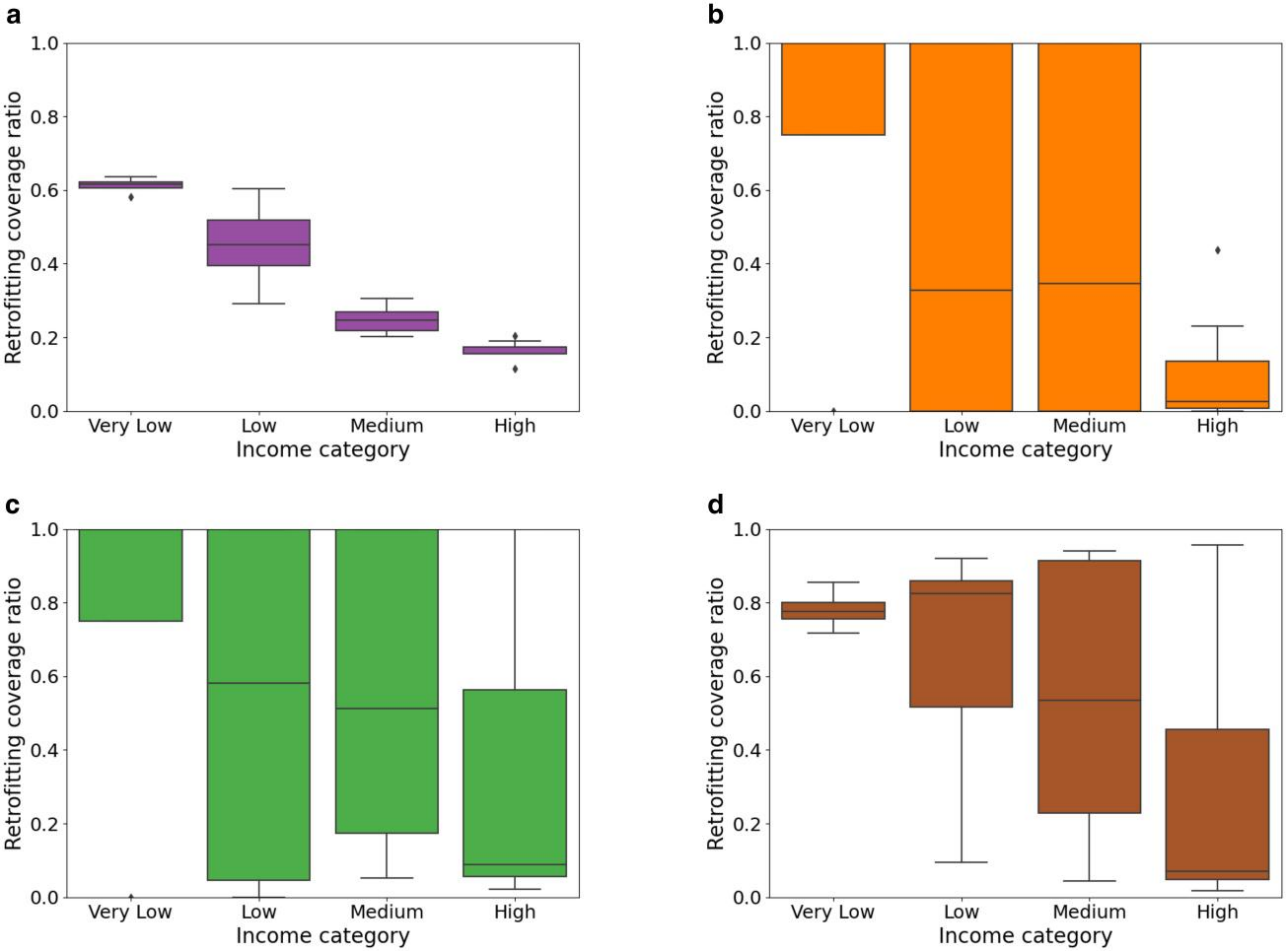
Retrofitting coverage ratio in counties of different income categories, for different ${\text{IW}}_{\epsilon }$ index in RAISE: The box plots present the summary statistics of the retrofitting coverage ratio across different income categories of the counties. The counties are categorized into very low, low, medium, and high based on their median income level. The retrofitting coverage ratio varies between 0 to 1. a) Baseline allocation to prioritize allocation to very low and low-income households. b) Low ${\text{IW}}_{\epsilon }$ index with the objective of maximizing savings. c) Medium ${\text{IW}}_{\epsilon }$ index with the objective of maximizing savings. d) High ${\text{IW}}_{\epsilon }$ index with the objective of maximizing savings. The median retrofitting coverage in RAISE is typically high for very low-income counties across different settings.

Next, we analyze the distribution of funds based on the income category of the county, as illustrated in Fig. 4. The counties are categorized into four groups: very low, low, medium, and high, based on their median income. This categorization follows a similar approach to that used for households and is described in Income classification under Supplementary information. The median of the retrofitting coverage is generally high for very low-income counties across various settings. While the low and medium levels of the ${\text{IW}}_{\epsilon }$ index allocate no budget to certain counties and fully retrofit others, the high ${\text{IW}}_{\epsilon }$ index ensures that most counties across all income groups are retrofitted. Generally, the median also rises with an increasing ${\text{IW}}_{\epsilon }$ index. For all equity settings of the ${\text{IW}}_{\epsilon }$ index, there is a range from 0 to 1 across the counties for the low-income category, indicating a wide variation in retrofitting coverage for this group. Ultimately, the policymakers can determine the pivot for a controlled distribution to meet their objectives of energy savings while promoting socioeconomic development across counties.

#### Household-level insights

**Figure 5:**
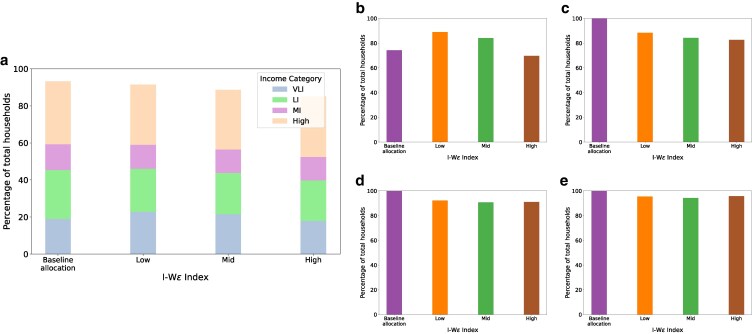
Percentage of households with retrofitting coverage ratio < 0.1 for various income categories for different ${\text{IW}}_{\epsilon }$ index in RAISE. a) Overall household percentage with low retrofitting coverage ratio when compared across different income groups. b) VLI household percentage with low retrofitting coverage ratio when compared with themselves. c) LI household percentage with low retrofitting coverage ratio when compared with themselves. d) MI household percentage with low retrofitting coverage ratio when compared with themselves. e) HI household percentage with low retrofitting coverage ratio when compared with themselves.

We compare the percentage of households of various income groups^c^ with low (<0.1) retrofitting coverage ratio, under different allocation strategies. As illustrated in Fig. 5a, RAISE, in comparison with the baseline allocation strategy, reduces the total percentage of households with inadequate retrofitting coverage with a higher reduction achieved for higher ${\text{IW}}_{\epsilon }$ index. Figure 5b–e further demonstrate that the baseline strategy allocates all funds to the VLI category. In contrast, RAISE, at low and medium ${\text{IW}}_{\epsilon }$ index, spreads a portion of the budget among other categories. While this reduces the total number of households with a low retrofitting coverage ratio, it incurs an increase in the percentage of VLI households when compared to the baseline allocation policy. Interestingly, RAISE with high ${\text{IW}}_{\epsilon }$ index is able to reduce the percentage of households with low retrofitting coverage ratios for all income categories in comparison to the baseline strategy. This, in addition to the impressive increase in energy savings described earlier, highlights the advantages of our tunable two-step approach.

Observation 2 RAISE with higher ${\text{IW}}_{\epsilon }$ index values lead to broader retrofitting coverage across counties.

Observation 3 The median retrofitting coverage increases generally with ${\text{IW}}_{\epsilon }$ across the different income categories.

### Evaluating RAISE across targeted population groups

In this next series of experiments, we focus on specific target groups for retrofitting rather than the entire population. We examine experiments aimed at different income levels, considering three target groups, of decreasing size: (i) LMI consisting of very low (VLI), low (LI), and medium (MI) income population group; (ii) V+LI consisting of very low (VLI) and low-income (LI) population groups; and (iii) VLI population consisting only of the very low-income population group. As described in Supplementary information, we categorize income levels based on guidelines from the Department of Housing and Urban Development (HUD) and other energy retrofitting programs.

**Figure 6:**
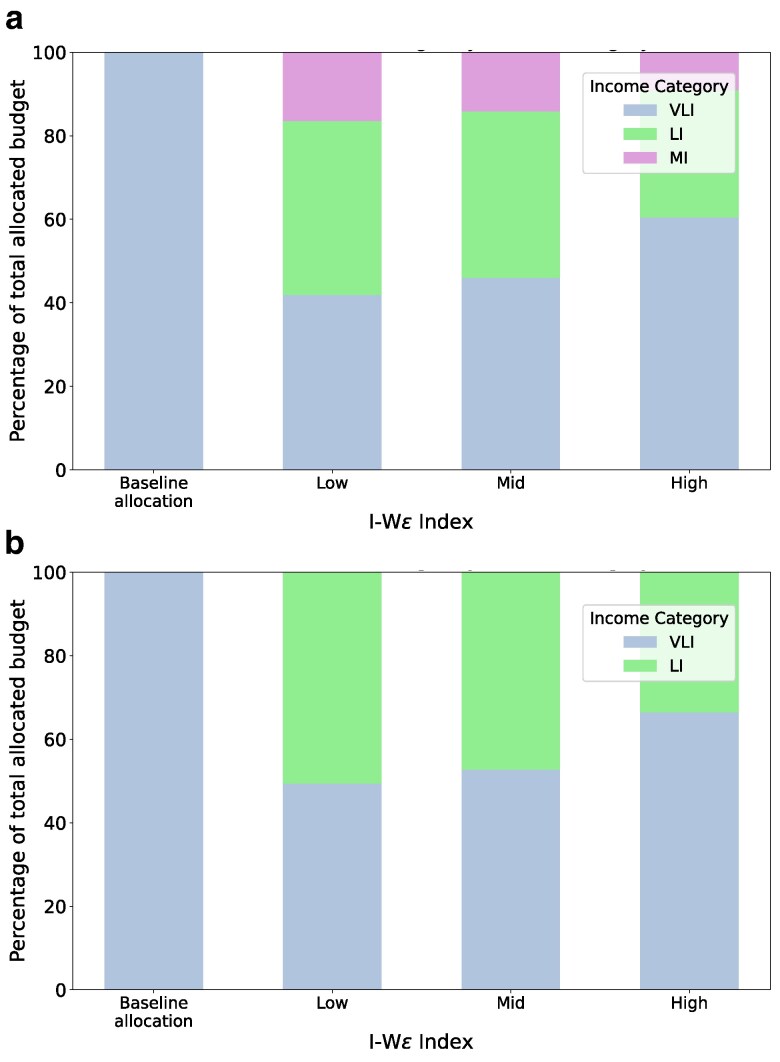
Share of allocated budget in targeted groups: a) Allocated budget share to each income category when only the LMI population is considered. This includes very low, low, and medium income categories. The *x*-axis consists of different strategies, and the *y*-axis is the share of allocated budget, a value between 0 and 1. b) Allocated budget share to each income category when only the V+LI population is considered. This includes very low and low-income categories. The axes are similar to the previous setup.

We begin by comparing the total allocated budget for each income category within the target group, as illustrated in Fig. 6. Note that the baseline allocation assigns 100% of its budget to very low-income households. For both the LMI and V + LI target groups, a rise in the ${\text{IW}}_{\epsilon }$ index in RAISE corresponds to an increasing share of the budget allocated to very low-income households. For lower ${\text{IW}}_{\epsilon }$ index values, the budget split between the very-low-income and low-income categories is nearly equal. However, as the ${\text{IW}}_{\epsilon }$ index rises, a portion of the budget from both the low-income and medium-income households is redirected to the very low-income category.

**Figure 7:**
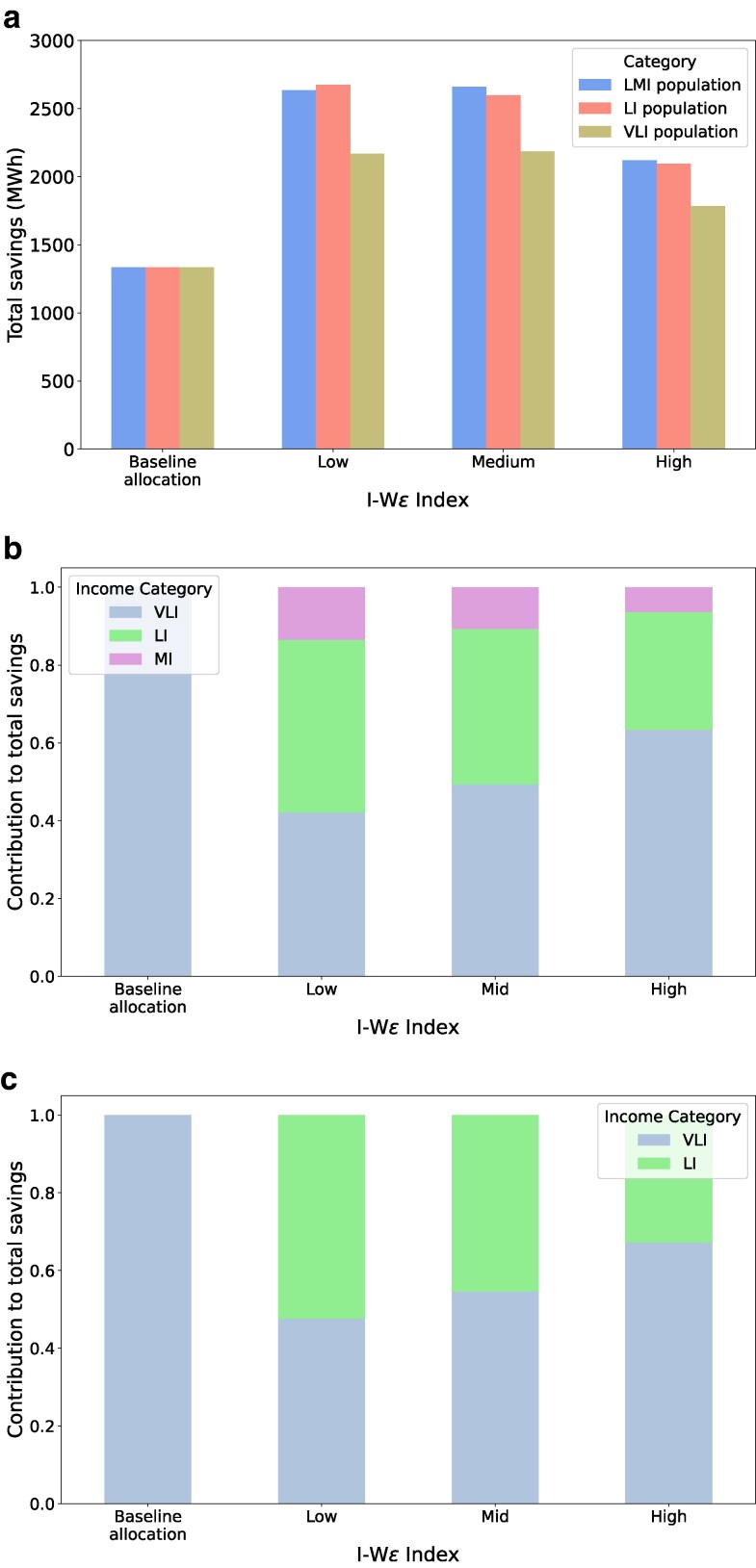
Total savings and contribution by targeted groups: a) Total savings by LMI, LI, and VLI population group. The *x*-axis represents the different strategies, and the *y*-axis represents the total savings in MWh. b) Contribution to total savings when only the LMI population is considered as target group. This includes very low, low, and medium income categories. The *x*-axis consists of different strategies, and the *y*-axis is the contribution to total savings, a value between 0 and 1. c) Contribution to total savings when only the V+LI population is considered as target. This includes very low and low-income categories. The axes are similar to the previous setup.

Next, we compare the total savings generated under each target group, as shown in Fig. 7a. Under different settings of the ${\text{IW}}_{\epsilon }$ index, the total savings generally increase when more categories of households are included in the target set. Interestingly, the difference in savings between the LMI and V+LI target groups is smaller than that between the V+LI and VLI groups. At low settings of the ${\text{IW}}_{\epsilon }$ index, the total energy savings of the V+ LI target category slightly exceed those of the LMI population.

Finally, we analyze the relative energy savings of different income categories under the allocation for targeted groups. The baseline’s entire savings arise from VLI households as allocation is directed solely towards the VLI income category. For the LMI target group illustrated in Fig. 7b, the savings contributed by the medium-income households are significantly less than savings from the VLI and LI households. Further, as the ${\text{IW}}_{\epsilon }$ index is made higher, the proportional contribution of energy savings from the VLI households increases as more of the budget allocation is directed towards them. In experiments for the V+LI target group depicted in Fig. 7c, the contributions are divided between the low and very low-income groups, with a primary emphasis on the latter. More experimental results are given in Supplementary information. We also performed a similar analysis on the contributions of different income categories and the budget allocated to each category under RAISE for the entire population in Supplementary information.

Observation 4 With an increase in ${\text{IW}}_{\epsilon }$ index, RAISE results in an allocation that increasingly favors the very low-income population even when different target populations are considered.

Observation 5 For all three settings of the ${\text{IW}}_{\epsilon }$ index, the total savings significantly increase for V+LI targeted group compared to the VLI group. The energy savings only slightly increase for the LMI target group over the V+LI target group.

## Methods

RAISE comprises two main components: (i) a retrofitting framework, which involves creating a synthetic model of residential energy demand to develop scenarios for energy usage before and after retrofitting at the household level and (ii) an optimization framework that models budget allocation to maximize savings while ensuring an income-weighted fair allocation using the ${\text{IW}}_{\epsilon }$ index. A summary of the notations used, along with their descriptions, can be found in Supplementary information.

### Data sources

In this work, we have used following datasets: (i) synthetic population data for VA (38) to capture the household information and their demographic characteristics and (ii) household pre-retrofitting and postretrofitting data (32) modeled using the residential energy demand framework (39).

### Retrofitting framework

First, we utilize the retrofitting scenarios modeled in the literature by Kishore et al. (32) using the energy demand framework developed by Thorve et al. (39) for the synthetic population (38). To provide a clear understanding, we present a brief overview of the energy demand modeling framework and the resulting synthetic dataset.

The energy demand modeling framework (39) utilizes realistic synthetic populations along with a variety of surveys and datasets to create detailed energy use profiles at the household level, generating hourly data. These data are broken down into two categories: thermostatically controlled loads (TCL) and appliance usage, modeled using a bottom-up approach. The framework incorporates individual appliance models that simulate the hourly energy consumption of various end uses, including heating, ventilation, and air conditioning (HVAC), lighting, refrigerators, hot water systems, and major appliances such as dishwashers, clothes washers, clothes dryers, and miscellaneous plug loads. Hourly energy use is calculated for each appliance, taking into account household occupancy and behavior. The synthetic energy demand data have been thoroughly validated across the entire United States. A comprehensive overview of the energy demand modeling framework is provided in the Supplementary information.

Kishore et al. (32) extended this framework to simulate three retrofitting scenarios: (i) upgrading insulation by increasing the R-values for ceiling and walls to meet the standard values, (ii) upgrading lighting to LEDs that are the most energy-efficient lighting appliances compared to compact fluorescent lamp (CFL) and incandescent bulbs, and (iii) upgrading appliances such as dishwasher and laundry appliances to energy star based on energy star ratings.

We calculated the energy consumption before and after retrofitting for a specific day. The difference between pre-retrofit and postretrofit energy usage in a household represents the energy savings for that day. This simulation was conducted over 12 random days, one from each month, for the state of VA, which has 3 million households. Next, we averaged the energy consumption from these 12 days. This approach allows us to capture the diversity in energy consumption resulting from seasonal variations. The resulting energy savings from the pre- and postretrofit energy use data generated from the energy demand modeling framework and the corresponding retrofitting costs are then provided as input into the optimization framework.

### Optimization framework

Our goal is to allocate a retrofitting budget to maximize cost savings while ensuring a balanced allocation across counties and within counties weighted by household income. We first define parameters for households and counties and the ${\text{IW}}_{\epsilon }$ index.

Definition 1 **(Parameters)**

We consider a state with *M* counties and *N* households. Each household *i* has the following attributes: the retrofitting cost (price) $p_{i}$ (in dollars) needed to require to retrofit the house; the energy savings $s_{i}$ (in kWh) achieved through retrofitting, where $p_{i},s_{i}>0$; and weight $w_{i}$ based on the household’s income. The $k^{\mathrm{th}}$ county has a county-weight $W_{k}$ based on the median income of households in it. We use $C_{k}$ to represent the set of households in $k^{\mathrm{th}}$ county. The household allocated budget is denoted as $x_{i}$ for $i^{\mathrm{th}}$ household and $y_{k}$ for $k^{\mathrm{th}}$ county.

The ${\text{IW}}_{\epsilon }$ index is derived based on ϵ-fairness, introduced in Ref. (40) based on a convex relation between 1 and 2 norms.

Definition 2 **(${\text{IW}}_{\epsilon }$ index)**

For a nonnegative vector $x\in R^{n}$ of dimension *n*, and parameter ϵ∈[0,1], *x* is said to be at least *weighted*  ϵ*-fair* (40, 41) with respect to a set of nonnegative weights $w\in R^{n}$ if $(1-\epsilon +\epsilon \sqrt{n})\cdot$  $\|w\circ x{\|}_{2}\leq \|w\circ x{\|}_{1}$. Correspondingly, we say that *x* satisfies the ${\text{IW}}_{\epsilon }$ index with weight *w* and parameter ϵ if

(1) $$F(x,w)\geq \epsilon \;\equiv \;\frac{\|w\circ x{\|}_{1}-\|w\circ x{\|}_{2}}{(\sqrt{n}-1)\|w\circ x{\|}_{2}}\geq \epsilon .$$

Note that ϵ=0 corresponds to the most unconstrained and *unfair* distribution of values in *x*, while ϵ=1 represents the most constrained *fair* distribution. We now formulate the budget allocation problem with ${\text{IW}}_{\epsilon }$ index below.

Problem 1 **(Budget allocation with ${\text{IW}}_{\epsilon }$)**

Given a total allocated retrofitting budget B, distribution parameter ϵ, details of all households and counties mentioned in Definition 1, determine the allocated budget $x_{i}$ for $i^{\mathrm{th}}$ household and $y_{k}$ for $k^{\mathrm{th}}$ county to maximize total energy savings, such that $x_{i}$s, $y_{k}$s satisfy the ${\text{IW}}_{\epsilon }$ index, where $x_{i}\in R_{\geq 0}$ and $y_{k}\in R_{\geq 0}\;\forall \;i,k$, within county and across counties. It is formalized as:

(Problem 1) $$\max_{x,y}\;\sum_{k=1}^{M}\sum_{i\in C_{k}}x_{i}\cdot \frac{s_{i}}{p_{i}}$$

(2) $$\text{s.t.}\;y_{k}=\sum_{i\in C_{k}}x_{i}\;\forall \;1\leq k\leq M$$

(3) $$0\leq x_{i}\leq p_{i}\;\forall \;1\leq i\leq N$$

(4) $$\sum_{k=1}^{M}y_{k}\leq B$$

(5) F(y,W)≥ϵ,

(6) $$\forall \;k,F(x_{C_{k}},w_{C_{k}})\geq \epsilon$$

(7) $$x_{i}\in R_{\geq 0},y_{k}\in R_{\geq 0}\;\forall \;i,k$$

In Problem 1, (3) and (4) represent constraints on individual households and total budget, respectively. (5) and (6) impose ${\text{IW}}_{\epsilon }$ index (see Definition 2), for budget allocations between counties, and between households in each county, respectively. Note that ${\text{IW}}_{\epsilon }$ index between counties uses county-weights $W_{k}$s, that are based on the median income of households in each county. Here, $x_{C_{k}},w_{C_{k}}$ denotes entries of x,w in $C_{k}$.

**Two-level solution approach**: Problem 1 is convex as the ${\text{IW}}_{\epsilon }$ index constraints (5), (6) are second-order cones (SOC). As the constraints (6) are separable into counties, for computational efficiency, we decompose Problem 1 into two levels (state and county) and iteratively solve them. The first, state-level optimization problem (P1), allocates budget among counties, based on their average saving rates $r_{k}$ (kWh/$), and ${\text{IW}}_{\epsilon }$ index using the county weights $W_{k}$. The second, county-level optimization problem (P2), distributes the budget allocated to each county among its households to maximize savings and imposes ${\text{IW}}_{\epsilon }$ index with household weights for that county. Note that (P2) for each county can be solved in parallel, enabling a computational speed up. The average saving rates $r_{k}$ for the state-level problem is estimated first as $r_{k}=\frac{\sum_{i\in C_{k}}s_{i}}{\sum_{i\in C_{k}}p_{i}}$. After the first iteration, we update the county saving rates $r_{k}$ with the ratio of realized savings to the budget allocated among households $C_{k}$ in the respective counties. We iteratively solve the state and county-level problems with rate updates till the total expected energy savings at each stage converge.

**State level problem:**


(P1) $$\max_{y}\;\sum_{k=1}^{M}y_{k}r_{k}$$

(8) $$\text{s.t.}\;\sum_{k=1}^{M}y_{k}\leq B,\;\forall \;k,y_{k}\leq \sum_{i\in C_{k}}p_{i},$$

(9) F(y,W)≥ϵ

(10) $$y_{k}\in R_{\geq 0}\;\forall \;k$$

**County level problem:** For kth county,


(P2) $$\max_{x_{C_{k}}}\;\sum_{i\in C_{k}}x_{i}\cdot \frac{s_{i}}{p_{i}}$$

(11) $$\text{s.t.}\;\sum_{i\in C_{k}}x_{i}\leq y_{k},\;0\leq x_{i}\leq p_{i}\;\forall \;i\in C_{k}$$

(12) $$F(x_{C_{k}},w_{C_{k}})\geq \epsilon$$

(13) $$x_{i}\in R_{\geq 0}\;\forall \;i$$

**Single-level formulation (alternate):** A different single-level version of Problem 1 can be formulated where the ${\text{IW}}_{\epsilon }$ index for households within each county (see (6)) is replaced with a single equity constraint for all households in the state. Aside from being computationally prohibitive, this can lead to infeasibility as a constraint involving all households in the state may not obey the constraint among budgets between counties, listed in (5). Our two-level approach to Problem 1 overcomes both these issues. Note that the constraints of the ${\text{IW}}_{\epsilon }$ index vary between the two-level and the alternate single-level formulations. These differences are due to the fact that ${\text{IW}}_{\epsilon }$ index is influenced not only by the value of ϵ but also by the population size. While both approaches produce similar savings, as demonstrated in Table S3, adjusting ϵ between the two formulations can help minimize discrepancies further. Additionally, the two-level model is favored for its greater computational efficiency. Additional algorithmic details to efficiently navigate the nonlinear, convex constraint for the ${\text{IW}}_{\epsilon }$ index, are detailed in Supplementary information under Algorithms/computational results. The Supplementary information also includes computational results for the alternative of Problem 1 for VA (3 million household variables), which justifies our problem formulation and solution approach.

## Conclusion

As energy demand proliferates, it has become essential to upgrade homes that are not energy-efficient. Retrofitting not only reduces the financial burden on residents by lowering their utility bills but also helps alleviate strain on the electrical grid. While the rationale for retrofitting is evident, determining the best candidates for such upgrades remains a key challenge. In this research, we proposed a novel framework RAISE for retrofitting budget allocation that maximizes energy savings while guaranteeing a balanced allocation across households in different counties and income groups, based on a tunable ${\text{IW}}_{\epsilon }$ index.

The ${\text{IW}}_{\epsilon }$ index-based allocation in RAISE is an essential tool for policymakers, as it enables them to concentrate on their primary objective of Return on Investment of energy savings while ensuring income-based and spatial equity in allocation. Our findings indicate that ${\text{IW}}_{\epsilon }$ index-based optimization can lead to 60–101% increase in savings over current baselines. While the baseline allocation method primarily directs resources to very low-income groups, the ${\text{IW}}_{\epsilon }$ index effectively distributes resources to various population segments at different income levels. Furthermore, experiments focusing on income-targeted populations revealed that concentrating on LMI groups yields savings almost equivalent to those achieved across the entire population when the total allocated budget is lower.

## Future research directions

Future extensions of the RAISE framework offer several promising directions. First, a key consideration for real-world implementation is how policymakers might select the equity parameter, ${\text{IW}}_{\epsilon }$. In our framework, this parameter is not prescriptive but serves as a decision-support tool that makes the trade-off between aggregate energy savings and distributional equity explicit. In practice, identifying an appropriate value of ϵ will likely require both quantitative calibration—such as benchmarking against historical allocation patterns—and qualitative stakeholder engagement through structured deliberation with utilities, community representatives, and retrofit providers. This participatory process parallels other policy domains with tunable funding weights (eg CDBG or LIHEAP).

Second, RAISE can be extended to multiobjective optimization, jointly considering goals such as maximizing energy savings, reducing carbon emissions, or ensuring minimum retrofit coverage for older or health-vulnerable housing stock. Because the ${\text{IW}}_{\epsilon }$ index functions as a tunable equity constraint, it can be readily integrated with additional objectives without altering the core structure of the model.

Third, expanding the framework to multiyear planning represents a natural next step, particularly under scenarios of constrained or declining federal support. Iteratively applying RAISE over successive budget cycles—with updated data on household eligibility, participation, and retrofit costs—would enable analysis of cumulative impacts, long-term coverage, and the evolution of equity outcomes over time.

Finally, while our present work focuses on income-based weights and a single-state case study in VA, future research could explore a broader set of geographic and demographic factors—such as housing type, construction period, or ownership status—in defining the ${\text{IW}}_{\epsilon }$ index. Similarly, relaxing the assumption of a uniform ${\text{IW}}_{\epsilon }$ constraint across state and county levels could reveal how different equity objectives affect the relationship between local and statewide allocations. Applying RAISE in other state or national contexts would also provide valuable insights into its generalizability and policy relevance.

## Supplementary Material

pgag162_Supplementary_Data

## Data Availability

The data and code underlying this article is available in UVA Dataverse (42) and can be accessed with doi 10.18130/V3/BHIB7J.

## Notes

a higher ϵ leading to a more balanced/fairer distribution

b Twice of the present funding proposed for residential energy efficiency programs in VA, but is attuned to the rising energy demand and corresponding grid stress.

c each income category (VLI, LI, MI, and High) represents a mutually exclusive group of households.
