# Supplementary material for: Tunable and computationally efficient framework for retrofitting: Budget allocation using income and spatial equity
Source: PNAS Nexus. 2026 May 12;5(5):pgag162. doi: 10.1093/pnasnexus/pgag162 (PMC13196871; doi:10.1093/pnasnexus/pgag162)
Supplement: pgag162_Supplementary_Data [file pgag162_supplementary_data.pdf]

Supplementary Information (SI)

Literature review

Retrofitting: Residential and commercial retrofitting literature explores various areas such as: (*i*) cost and energy optimization, (*ii*) environmental impact and emission reduction, (*iii*) indoor air quality and occupant comfort, (*iv*) occupant behavior, (*v*) policy and regulatory frameworks, and (*vi*) affordability and income-based strategies. While cost-based optimization focuses on reducing upfront costs, shortening the payback period, and minimizing life-cycle costs, energy optimization aims to reduce energy consumption by improving building components and integrating energy-efficient technologies.

Hashempour et al. [14] performed a literature review of on energy performance optimization of existing buildings. The review results indicate that, despite extensive studies conducted on retrofitting, certain objectives—especially those focusing on comfort conditions—have been neglected. Additionally, there is a need to develop a new decision-making tool that can reduce the computational time for optimization while improving the reliability of the optimal solutions. Dabous et al. [11] conducted a holistic review of building envelope retrofit techniques and evaluated their combined effects on energy efficiency, aesthetics, and indoor environmental quality.

Verbeeck et al. [35] highlighted the significance of retrofitting existing housing to reduce energy consumption. The study explored the optimal balance between costs and benefits, ultimately proposing a hierarchy for energy savings during retrofitting. Ferrara et al. [12] introduced a simulation-based optimization approach utilizing TRNSYS and GenOpt to identify cost-optimal retrofit configurations for single-family homes in France. This research identified building configurations that simultaneously minimize lifecycle costs and achieve nearly zero energy consumption. Mejjauli et al. [27] developed a decision-making model using Mixed Integer Linear Programming to select the most effective energy retrofitting strategies aimed at minimizing the life-cycle cost of buildings. The model's constraints included thermal comfort, illumination, and budget considerations. Luddeni et al. [25] proposed a simulation-based methodology to analyze and evaluate retrofitting measures for office buildings. The authors combined energy modeling with optimization techniques to identify cost-effective retrofitting measures for a variety of building stocks using 30 unique building reference models. Shu et al. [31] proposed a decision-making framework to identify robust energy retrofit measures for residential communities under varying conditions. They emphasized optimizing energy performance while accounting for cost-effectiveness and uncertainity.

Zhou et al [39] conducted a comprehensive case study to identify effective strategies for enhancing the energy efficiency of buildings. The retrofitting measures implemented in the case buildings included improvements to building envelopes, HVAC systems, and lighting. The findings highlight the significant potential of targeted retrofitting to reduce energy consumption and operational costs effectively. Sharma et al [30] focused on evaluating various retrofit strategies using a calibrated simulation approach. They performed an energy performance audit and conducted year-long monitoring of an existing building to calibrate their simulation model and assess the impact of basic energy conservation measures. Their results indicated that targeted retrofitting can significantly enhance energy efficiency, reduce operational costs, and improve occupant comfort. Li et al. [22] presented a systematic approach to improving energy efficiency in existing residential buildings constructed between 1980 and 2000. The authors proposed a design method that integrates facade system information modeling, which aids in developing comprehensive retrofitting plans tailored to different building typologies.

Multiple studies have highlighted the importance of retrofitting in minimizing the environmental impact of buildings. In their research, Ascione et al. [4] developed a multi-objective optimization framework using EnergyPlus to identify the best building envelope strategies for nearly zero-energy buildings (nZEBs) in Mediterranean climates. Their findings emphasized the challenge of balancing energy demands for summer and winter while ensuring energy comfort, as they evaluated the results across four different cities. Kolokotsa et al. [20] provided a review of technologies and their integration. They offered a broader systems-level perspective that combines renewable energy technologies, smart control systems, and demand-side management as components of an environmental strategy.

Literature on retrofitting emphasizes achieving multiple objectives, such as minimizing energy consumption, reducing retrofitting costs, and lowering carbon dioxide emissions, to name a few. Kadric et al. [19] tackled the challenge of balancing energy savings, carbon dioxide emissions reduction, and retrofit costs in residential buildings in Bosnia and Herzegovina. They identified pareto-optimal solutions by using a combination of full factorial design and the NSGA-III optimization framework to evaluate various retrofit strategies based on national building data. Their results indicate that upgrading external walls and heating systems are the most effective methods for reducing emissions and energy consumption, although these upgrades result in higher costs. Asadi et al. [2] proposed a comprehensive framework to help stakeholders identify effective retrofit interventions for existing buildings. Their model combined dynamic energy simulations with a Tchebycheff-based multi-objective optimization algorithm. This integration allowed for balancing conflicting objectives, such as minimizing energy consumption and retrofit costs, while also ensuring occupant comfort. Ascione et al. [3] proposed a methodology to improve the energy efficiency of educational buildings. A multi-objective optimization framework is utilized on energy performance data to identify retrofit strategies that balance energy consumption, thermal comfort, and economic factors.

There is also literature that emphasizes income-based retrofitting and its policy relevance. Willand et al. [37] examined the implications of distributional justice related to a market-based retrofit subsidy program. Their findings revealed that lower-income and renter-dense areas received significantly fewer benefits, suggesting that non-targeted subsidies may reinforce energy inequalities. Perez et al. [28] proposed a prioritization map to address energy poverty in social housing. This map identifies the most energy-vulnerable dwellings based on building characteristics and socioeconomic conditions. Additionally, Liang et al. [23] provided empirical estimates of the net energy savings from building energy retrofits in both residential and commercial buildings in Phoenix. Based on their study, they estimated how the effectiveness of retrofitting varies with different building attributes.

Martin et al. [26] proposed a method for retrofitting community buildings to help France achieve its goal of reducing greenhouse gas (GHG) emissions by a factor of ten. This plan involves retrofitting 50% of community structures. Additionally, Boza-Kiss et al. [7] conducted a comparative analysis of eight building energy efficiency policy instruments, highlighting their potential for achieving cost-effective energy savings. The study concluded that the overall effectiveness of these instruments depends on their design, implementation, and enforcement within specific contexts. A study by Zhang et al. [38] aimed to assist policymakers and other stakeholders in gaining a comprehensive understanding of retrofit policy instruments and their implementation. This research helped to identify barriers to the uptake of retrofit schemes and to develop more efficient retrofit policy instruments in the future.

There are also several research focusing on occupant behavior, air quality and occupancy comfort. Lozinsky et al. [24] performed a review of residential building envelope retrofits and found strong evidence for improved acoustic comfort, enhanced winter thermal comfort, and

reduced respiratory and cardiovascular health issues. Che et al. [10] highlighted the combined benefits of HVAC retrofitting in improving both energy efficiency and indoor environmental quality. This upgraded system, including sensor-based controls and enhanced filtration, reduced particle infiltration by 30–60% and maintained thermal comfort across seasons. Alazazmeh [1] demonstrated that implementing retrofitting measures reduced the building's energy load by 39% and significantly enhances indoor environmental quality alongside energy savings.

*Energy Justice:*A growing body of U.S. energy justice literature situates energy efficiency, housing, and retrofitting within broader issues of equity and social vulnerability. Research by Reames [29] and Bednar et al. [5] demonstrated that spatial, racial, and socioeconomic disparities affect access to energy-efficient housing, with low-income and minority households often living in older, less efficient buildings. Hernández [15, 16, 17] conceptualized energy insecurity as a multidimensional challenge involving affordability, housing quality, and health. The author highlighted how disconnections from energy services and inadequate investments in efficiency exacerbate existing inequalities. Complementary analyses, such as those by Goldstein et al. [13] and Lewis et al. [21], confirmed the persistent racial gaps in household energy efficiency and emissions, emphasizing the need to view energy efficiency as a justice-driven intervention. Additionally, reviews and policy analyses have contributed to a systemic perspective on energy justice. Carley and Konisky [9] provided a comprehensive synthesis of environmental policy literature, identifying energy justice as a rapidly expanding field. Bulkeley et al. [8] discussed how urban climate action must evolve to incorporate climate equity through inclusive governance and distributional justice. Sovacool et al. [32] evaluated the justice and equity dimensions in low-carbon energy transitions. Jenkins et al. [18] proposed strategies for embedding energy justice principles across clean energy research and development in the U.S.

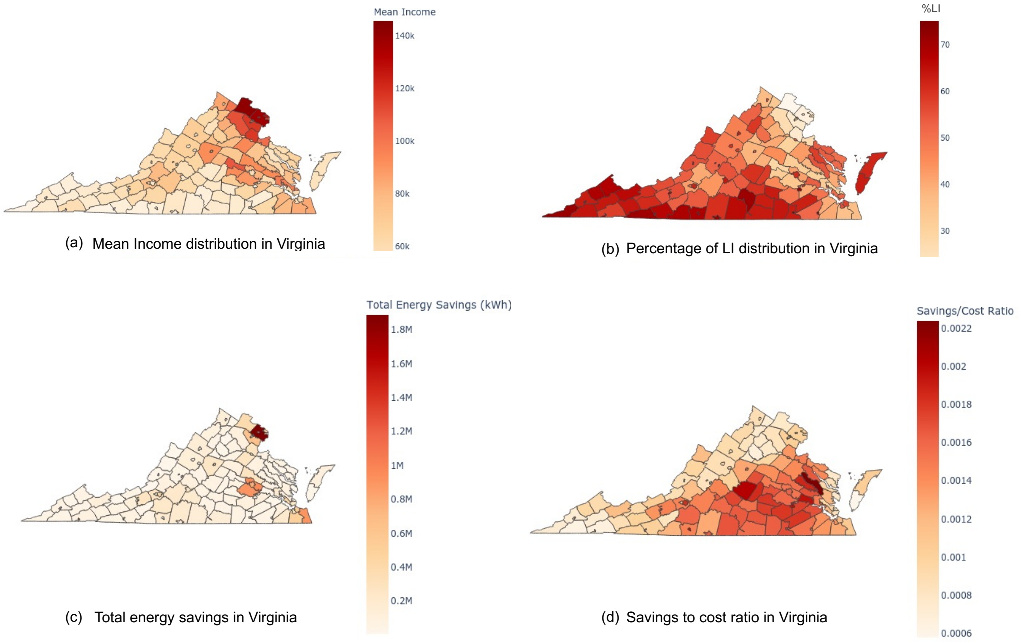

(a) Mean Income distribution in Virginia

(b) Percentage of LI distribution in Virginia

(c) Total energy savings in Virginia

(d) Savings to cost ratio in Virginia

Fig. 1: **Virginia statistics based on data**: (a) Mean income distribution for each county in Virginia. (b) LI percentage distribution for each county in Virginia (c) Total energy savings for each county in Virginia (d) Savings to cost ratio for each county in Virginia.

Virginia statistics

We present various statistics for Virginia in Figure 1. Figure 1a illustrates the mean income distribution across each county in Virginia. It is evident that counties in northern Virginia have disproportionately high income levels compared to others. In contrast, central Virginia, which is primarily rural, shows a comparatively lower mean income. Figure 1b depicts the percentage of the low-income (LI) population in each county. This plot resembles a mirror image of the first one. Figure 1c displays the total energy savings for each county in Virginia, with the three populous urban counties contributing the most to energy savings. Figure 1d represents the savings-to-retrofitting cost ratio for each county. Generally, this ratio appears to be higher in the central, piedmont, and eastern regions of Virginia.

Income classification

The income categories are defined as Very Low Income (VLI), Low Income (LI), Medium Income (MI), and High Income, based on household income relative to the area median income (AMI) of the state. According to the income categorization provided by the Department of Housing and Urban Development (HUD) and other retrofitting energy programs, households with a budget of less than \$30,000 are classified as VLI, which represents approximately 30-40% of the AMI. Households with a budget below \$60,000 and above \$30,000 are considered LI, accounting for about 50-60% of the AMI. Households with a budget below \$80,000 and above \$60,000 are categorized as MI, representing approximately 80-120% of the AMI. Households with incomes above this threshold are classified as High Income. We also classify counties into income groups. For this classification, we utilize the same income categories as households but base it on the county's median income.

Selection criteria

We select the households that have household income less than $200,000$ [36] dollars and a daily savings-to-retrofitting cost ratio of at least 0.002. Based on Virginia's average electricity price ($0.013/kWh$), an annual Savings-to-Investment Ratio (SIR) of greater than one over a 15-year lifespan at a 4% discount rate is required to fund for retrofitting [34]. Choosing a value of the daily savings-to-retrofitting cost ratio of 0.002 helps to achieve this.

Sensitivity analysis using different energy savings settings

We conducted a sensitivity analysis across different energy consumption settings. The results show that trends remain consistent across the three IW_ϵ index values. In addition, we tested robustness by cross-multiplying energy profiles from one setting (e.g., maximum consumption) with budget allocations derived from another setting (e.g., average consumption), and vice versa. The results of this analysis are presented in Figure 2.

The total energy savings obtained from cross-multiplying budgets and energy profiles in average and maximum scenarios are very close across all three IW_ϵ index settings. The small observed differences arise primarily from slight variations in the number of eligible households between the two scenarios ($3,066,089$ for average consumption versus $3,067,352$ for maximum consumption).

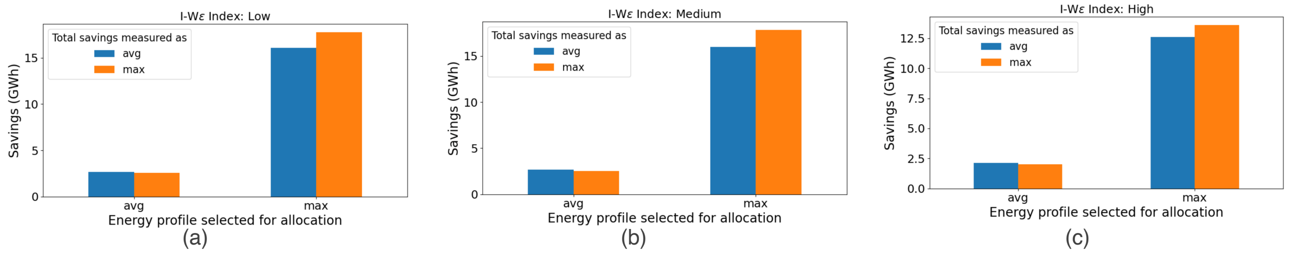

(a) (b) (c)

Fig. 2: **Sensitivity analysis for different criteria of energy profiles used for budget allocation, at various IW_ϵ Index in RAISE:** Total energy savings (measured in terms of average or maximum over the state) for two strategies that use respectively average and maximum energy consumption per household as criteria to determine budget allocations inside RAISE. The three plots consider different values of IW_ϵ Index: (a) low, (b) medium, and (c) high. The small differences, across different ϵ values, show that the quantified total energy savings (both max and average) are generally insensitive to the specific household consumption criteria (max or average) used for budget allocation.

Energy savings and budget allocation

Figure 3 illustrates the energy savings and budget allocations for various income categories under different settings of the IW_ϵ Index. It is important to note that energy consumption is not directly proportional to the savings gained from retrofitting. Although high-income households may use more energy in absolute terms, they do not necessarily achieve proportionally greater savings from retrofits, as their potential for improvement tends to be limited. In contrast, low-income households often experience the most significant relative energy savings when retrofitted. As the IW_ϵ Index increases, the proportion of the budget allocated to the VLI group rises substantially, at the expense of the shares allocated to high and medium-income categories.

Energy demand modeling framework

The energy-demand modeling framework created by Thorve et al. [33] begins with a statistically representative synthetic population of U.S. households, derived from census microdata. This framework utilizes an open-source version of the U.S. synthetic population called Synthetic Populations and Ecosystems of the World (SPEW). The SPEW synthetic population includes demographic characteristics of both synthetic households and synthetic individuals. To ensure statistical accuracy in the synthetic population, sampling and Iterative Proportional Fitting (IPF) methods are used to maintain the accuracy of marginal distributions across various demographic categories. This approach guarantees statistical realism in the creation of the synthetic population.

The foundational population is enhanced with additional attributes related to energy and activity through machine-learning models that are trained on national energy survey data, such as the American Time Use Survey (ATUS) and the Residential Energy Consumption Survey (RECS). The resulting enriched synthetic households, along with publicly available datasets related to buildings, weather, and behavior, are used as inputs for a bottom-up residential energy use modeling system.

The framework addresses nine major end uses of energy in households: heating, cooling, lighting, domestic hot water, refrigeration, cooking, dishwashing, clothes washing and drying, and miscellaneous plug loads. These are categorized into thermostatically controlled loads (TCL) and appliance loads. The HVAC component estimates heating and cooling electricity requirements based on outdoor temperature and system efficiency. The domestic hot water model predicts energy usage for activities such as bathing, laundry, and dishwashing. The lighting model calculates power consumption according to occupancy and time of day. Refrigeration is

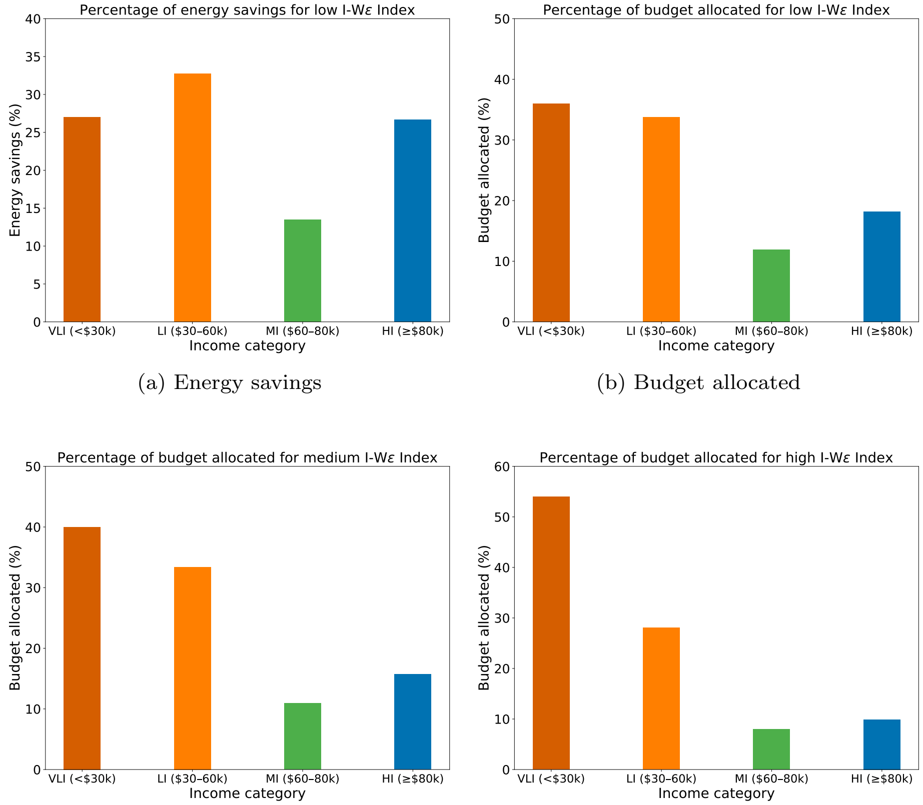

(a) Energy savings

(b) Budget allocated

(c) Budget allocated

(d) Budget allocated

Fig. 3: While high-income households consume more energy in absolute terms, they do not necessarily generate proportionally higher savings when retrofitted (Fig (a)), and hence not allocated high budget. This is illustrated by the figures provided (see Budget Allocation vs. Savings across Income Categories at Low, Medium, and High IW_ϵ settings in Fig (b), (c), (d)). For instance, under low fairness, very low and low-income households receive a substantial share of the budget ($\approx$ 70%) for their energy savings share($\approx$ 60%). As the fairness settings increase to medium and high, this share rises to about $\approx$ 75% and $\approx$ 83%, respectively. Conversely, the budget allocation for high-income households decreases from approximately $\approx$ 18% to around $\approx$ 9%.

modeled as a steady temperature-dependent load. The cooking energy model captures the use of electric ranges and kitchen appliances, while the models for other appliances account for dishwashers, clothes washers and dryers, as well as miscellaneous plug loads like televisions, computers, and cleaning devices. Together, these modules provide a detailed, household-level representation of residential electricity demand, incorporating demographic, behavioral, and climatic factors for realistic end-use simulations.

Additional insights on 1 billion dollar investment

Distribution of household allocations across counties

We analyze the impact of empirical IW_ϵ Index for the budget allocated by RAISE to Virginia's counties using different approaches. In this study, we use the empirical IW_ϵ Index to quantify the equity of each county's allocations to its households, and we present declining trends in equity over the set of counties in Figure 4. The baseline approach, which focuses on income-based budget allocation across the state, shows a consistent downward trend, starting from an equity score below 0.5. RAISE-based allocation are above the baseline for a majority of counties. In particular, RAISE with high IW_ϵ Index is strictly above the baseline aside for one county.

Furthermore, empirical slopes under RAISE derived from the three settings of the IW_ϵ Index are steeper than those observed with the baseline approach.

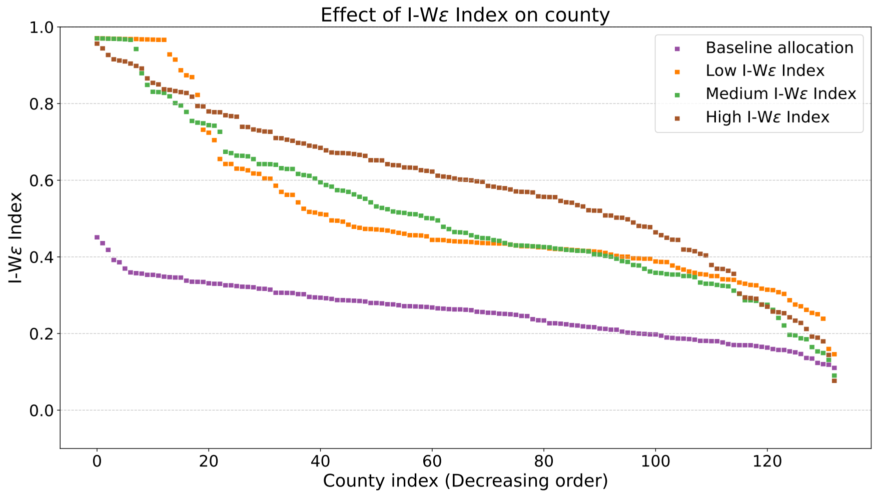

Fig. 4: **IW_ϵ Index comparison**: The x-axis shows the county index in decreasing order of the IW_ϵ Index, and the y-axis shows the value of the IW_ϵ Index. The purple strips represent the baseline very low and low-income allocation, and the orange represents the low IW_ϵ Index allocation, and the green and the brown represent the medium and high IW_ϵ Index, respectively.

Insights on savings and budget allocation

We analyzed the contributions of different income categories and the budget allocated to each category under RAISE, as shown in Figure 5.

Figure 5a shows that only the VLI category is chosen when using the income-based baseline allocation strategy. In contrast, RAISE allocates budgets to different income categories, with an increasing budget allocation to the VLI category as the I-Wϵ Index rises. Proportionally, the contributions to total savings follow a similar trend as the allocated budgets, as illustrated in Figure 5b. This analysis also demonstrates that while energy savings primarily come from the VLI group, they are not limited to this category alone. Savings also extend to other income categories.

The budget allocation among counties in Figure 6 mirrors the pattern seen in the retrofitting coverage ratio in Figure 3 of the main manuscript. The baseline approach allocates more of the budget to Fairfax, Richmond, and Virginia Beach for the baseline. In the two-level approach, a low I-Wϵ Index Index results in a high budget allocation for counties in the central-eastern and piedmont regions, while other regions receive minimal funding. As the I-Wϵ Index Index increases to medium levels, funding starts to be distributed to northern and western counties, although the budget for certain counties that yield comparatively lower savings is slightly reduced. At a high I-Wϵ Index Index, the budget distribution expands further, allowing more counties to receive funds for retrofitting.

Insights on targeted population group experiments

In this section, we provide further insights into the experiments conducted on income-based target population groups with RAISE. The population is divided into three groups: LMI group, V+LI group, and VLI group. First, we present the retrofitting coverage allocation for each income category within these population groups, as illustrated in Figure 7. In this setup, for each

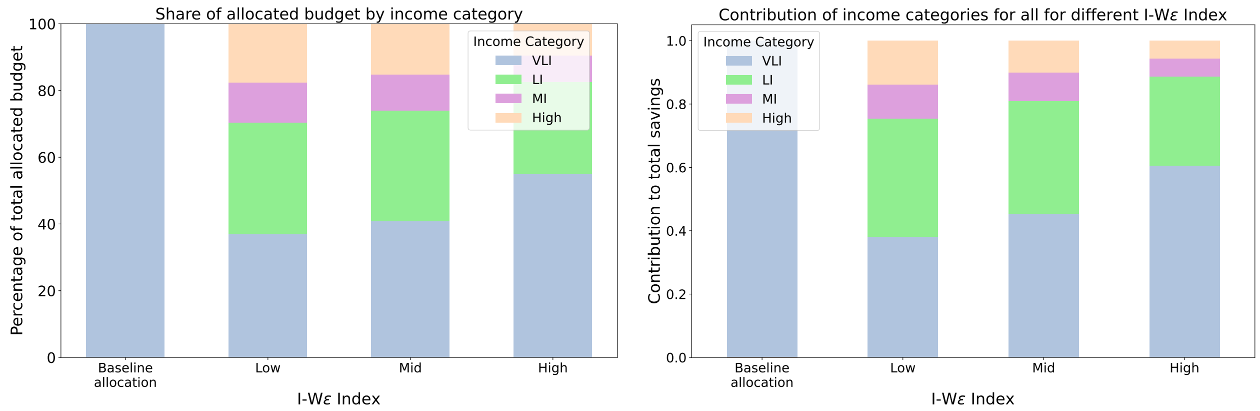

(a) Budget allocation (b) Contribution to savings

Fig. 5: **Savings contribution and budget allocation for 1 billion dollars.** The left panel shows the distribution of allocated budget across the same groups, while the right panel shows the relative savings contributions across income groups.

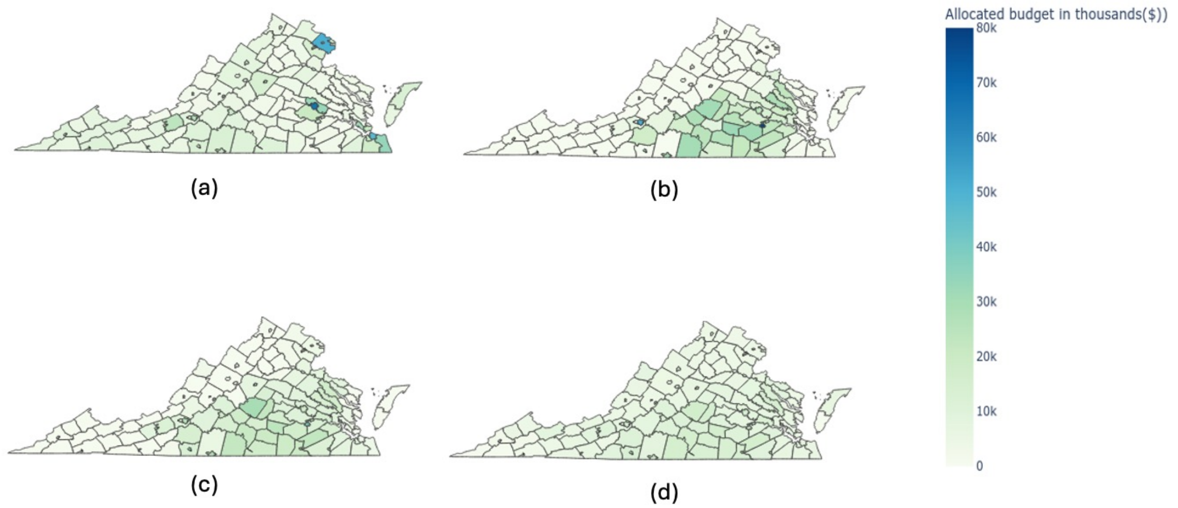

Fig. 6: **Allocated budget for different I-Wϵ Index in RAISE**: (a) Baseline allocation to maximize allocation to very low and low-income households. (b) Low I-Wϵ Index with the objective of maximizing savings. (c) Medium I-Wϵ Index with the objective of maximizing savings. (d) High I-Wϵ Index with the objective of maximizing savings.

income category, we compute the total retrofitting coverage it received, which is the distribution of the total allocated budget relative to the retrofitting cost for the particular income category.

The baseline strategy allocates its entire budget to the VLI category. While the shares of the VLI and LI categories are almost equal at low settings of I-Wϵ Index in RAISE in Figure 7a, the disparity increases in the LMI population group as the I-Wϵ Index settings rise. A similar trend is observed in Figure 7b for the LI population group, where the portion allocated to the medium income category shifts to the VLI category. The distribution of the VLI population, as shown in Figure 7c, indicates that any I-Wϵ Index settings in RAISE outperform the baseline allocation, with medium settings achieving the best performance, followed by high and then low settings.

Next, we present the percentage of households with a low retrofitting coverage ratio for allocations targeted to the LMI, V+LI, and VLI population groups. This is illustrated in Figure 8. For a specific target group (see Figure 8a, b or c), increasing I-Wϵ Index leads to

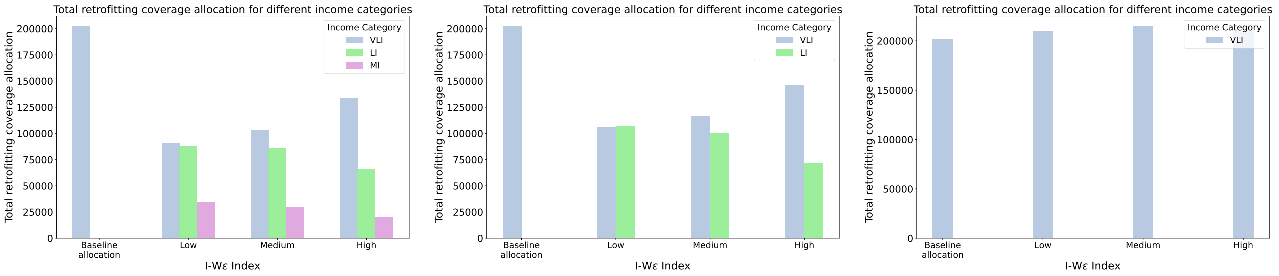

(a) Retrofitting coverage allocation to LMI (b) Retrofitting coverage allocation to V+LI (c) Retrofitting coverage allocation to VLI

Fig. 7: **Retrofitting coverage allocation for different income categories** The first panel shows the retrofitting coverage allocation for the LMI target group, the middle panel shows it for the V+LI target group, and the last panel shows it for the VLI targeted group. The retrofitting coverage ratio to the VLI income category increases as the IW_ϵ Index settings rise. In the VLI targeted group, the RAISE with IW_ϵ Index settings outperforms the baseline allocation, with the medium settings yielding the best results overall.

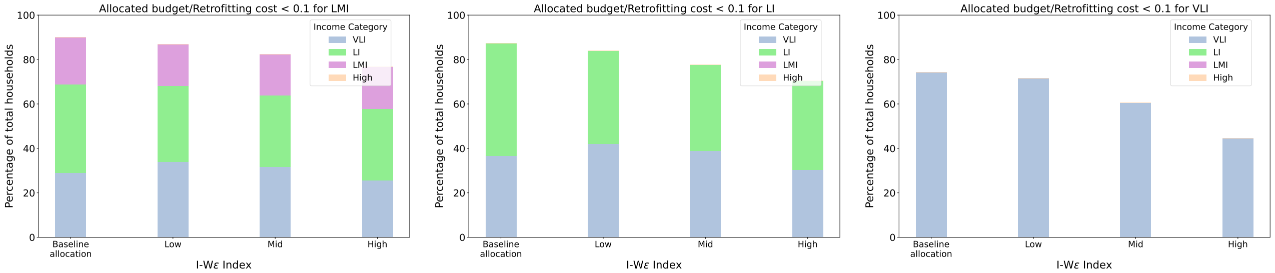

(a) LMI households (b) V+LI households (c) VLI households

Fig. 8: **Percentage of households with low retrofitting coverage in each population group.** The first panel shows the percentage of households for the LMI population group, the middle panel shows it for the V + LI population group, and the bottom panel depicts it for the VLI population group.

a reduction in the total percentage of households with a low coverage ratio due to the impact of the index. On the other hand, keeping the I-Wϵ Index fixed (low, medium, or high), the total percentage of households within the target group with a low coverage ratio goes down as the target group becomes more restrictive (LMI to V+LI to VLI). This is due to the limited allocation to a smaller group of households in restricted income groups. Notably, even when the allocation is directed solely towards the VLI population group, as shown in Figure 8c, the coverage ratio remains low for at least 50% of the houses. This indicates that a budget of \$1 billion is not sufficient for large-scale retrofitting, even when targeting the VLI group alone. However, compared to the baseline, RAISE-based allocation outperforms in energy savings and income distribution.

Analysis on higher budget investments

Total savings for higher budgets

We begin by presenting the total savings from various budget allocations based on the baseline allocation, along with different I-Wϵ Index values as shown in Table 1. RAISE demonstrates greater savings across various I-Wϵ Index settings for different budget levels. As the budget increases, the savings also tend to rise in an almost linear manner. In most scenarios, the medium settings outperform the other two equity levels, particularly in the budget range of 2 billion to 4 billion. However, at a budget of 5 billion, the medium equity settings show a slight decrease in savings, indicating diminishing returns.

Budget ($)	Baseline Allocation (kWh)	Low I-Wϵ Index (kWh)	Medium I-Wϵ Index (kWh)	High I-Wϵ Index (kWh)
1B	1,334,266	2,670,264	2,688,363	2,128,577
2B	2,638,725	4,593,785	4,687,013	3,774,093
3B	3,987,453	6,306,623	6,402,633	5,238,015
4B	5,334,497	7,834,033	7,935,065	6,506,675
5B	6,717,849	9,281,270	9,301,759	7,605,629

Table 1. Total allocated energy (in kWh) under baseline allocation and different I-Wϵ Index across increasing budgets.

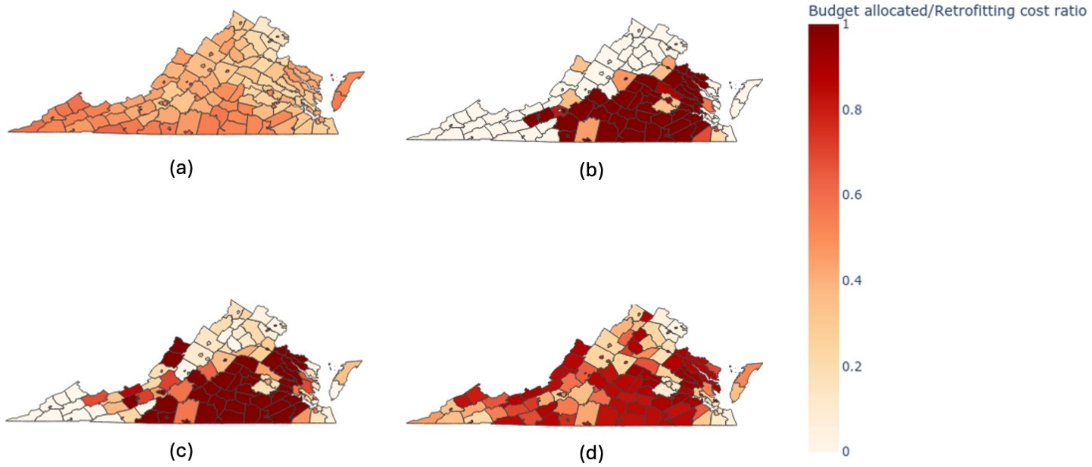

Fig. 9: **Retrofitting coverage ratio for different I-Wϵ Index in RAISE**:(a) Baseline allocation to maximize allocation to LMI households. (b) Low I-Wϵ Index with the objective of maximizing savings. (c) Medium I-Wϵ Index with the objective of maximizing savings. (d) High I-Wϵ Index with the objective of maximizing savings.

Next, we detail the results of different metrics for the budget allocation of 5 billion dollars (highest considered) in VA.

Retrofitting coverage ratio for 5 billion dollars

We now show the distribution of retrofitting coverage ratio for a budget of 5 billion dollars As the budget increases to 5 billion dollars, the baseline allocation will also include budgets for low-income households, in addition to very low-income households. The retrofitting coverage ratio is generally high when compared to the base experiments (budget of 1 billion dollars), as illustrated in Figure 9 compared against Figure 3 for 1 billion dollars in the main manuscript. In the low I-Wϵ Index setting, funding was concentrated in the central, eastern, and piedmont regions, while the high I-Wϵ Index settings ensured that funds were allocated to all counties in Virginia. It is noteworthy that despite having a high I-Wϵ Index, the retrofitting coverage ratio remains low in urban and densely populated areas, such as Fairfax, Loudoun County, Virginia Beach, and Richmond County. This metric measures the ratio of the allocated budget to the total cost of retrofitting. The requirement is typically high, given the large number of houses needing retrofitting due to population size compared to the available budget.

We compare the retrofitting coverage ratio across the counties, categorizing them into high, low, and medium-income as shown in Figure 10, similar to Figure 4 for 1 billion dollars in the main manuscript. Across all income groups, the average retrofitting coverage ratio tends to increase from low to medium to high levels of the I-Wϵ Index compared against the 1 billion

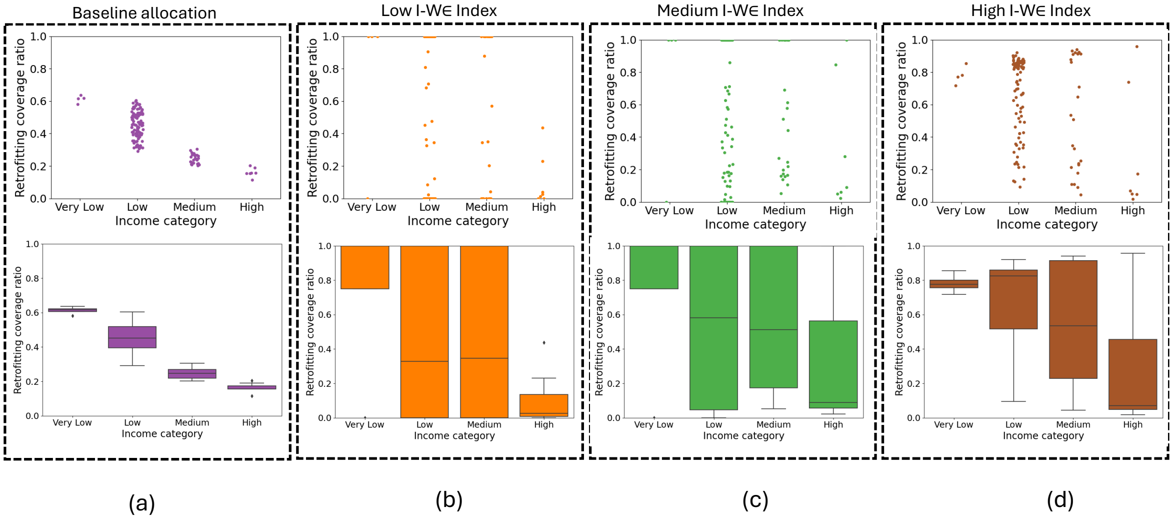

Fig. 10: **Retrofitting coverage ratio statistics comparison for different I-Wϵ Index in RAISE:** The top strip plots display the retrofitting coverage ratio on the y-axis for various income categories of the counties. The bottom box plots present the summary statistics of the retrofitting coverage ratio across different income categories of the counties. (a) Baseline allocation to maximize allocation to very low and low-income households. (b) Low I-Wϵ Index with the objective of maximizing savings. (c) Medium I-Wϵ Index with the objective of maximizing savings. (d) High I-Wϵ Index with the objective of maximizing savings.

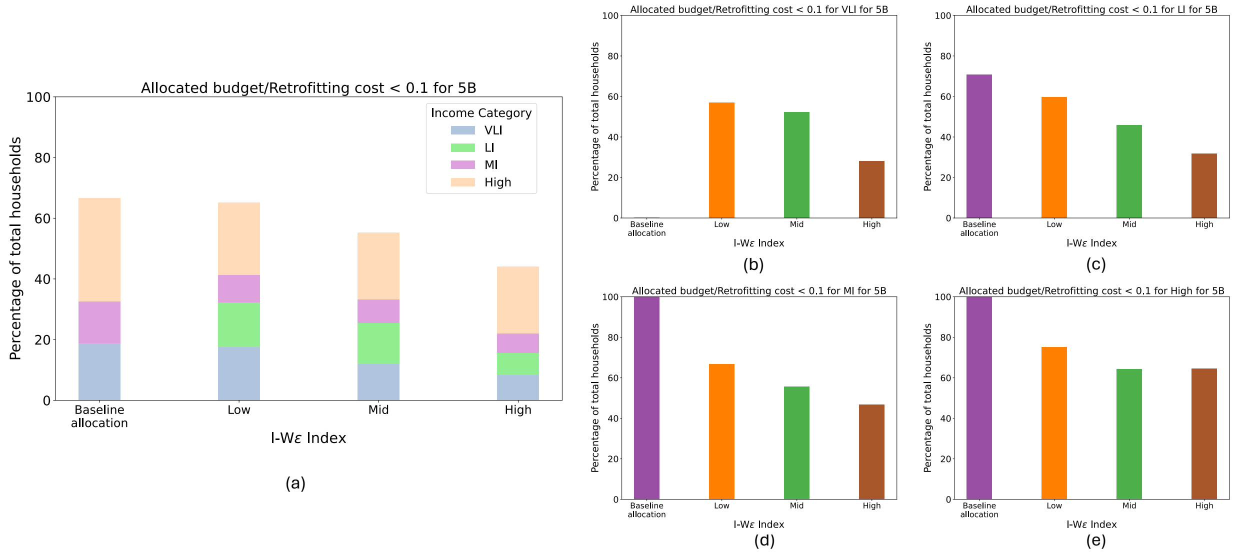

Fig. 11: **Percentage of households with retrofitting coverage ratio $<$ 0.1 for various income categories for different I-Wϵ Index in RAISE**: (a) Overall household percentage with low retrofitting coverage ratio when compared across different income groups. (b) Very low income (VLI) household percentage with low retrofitting coverage ratio when compared with themselves. Note that there are also almost no households in the VLI category for baseline allocation. (c) Low income (LI) household percentage with low retrofitting coverage ratio when compared with themselves. (d) Low-to-moderate income (LMI) household percentage with low retrofitting coverage ratio when compared with themselves. (e) High-income household percentage with low retrofitting coverage ratio when compared with themselves.

dollars. Generally, the median also rises with an increasing I-Wϵ Index, with the most significant rise occurring in the low and medium-income groups. This observation is similar to the budget allocation of 1 billion dollars. Next, we analyze the households with retrofitting coverage ratios less than 0.1 across different population groups as shown in Figure 11, similar to Figure 5 for 1

billion dollars in the main manuscript. The baseline approach represented in the figure indicates that there are no houses in the VLI category with low retrofitting coverage. In contrast, the I-Wϵ Index-based approach allocates funds to all income groups, resulting in representation from all four population groups across the three settings of the I-Wϵ Index. However, we observe that the low retrofitting coverage ratio in the very low, low, and medium income categories decreases as the I-Wϵ Index increases. Overall, the total percentage of households with low retrofitting coverage is lower in our approach compared to the benchmark. Initially, the baseline allocation for V+LI households only covered some houses in the VLI category with a budget of 1 billion dollars. However, when the budget increased to 5 billion dollars, approximately 30% of the houses in the LI category were included as well. Under various equity settings, there has been a significant number of houses retrofitted across all four income categories, with particularly notable reductions in the VLI, LI, and MI categories.

Algorithms/computational results

State-county savings convergence

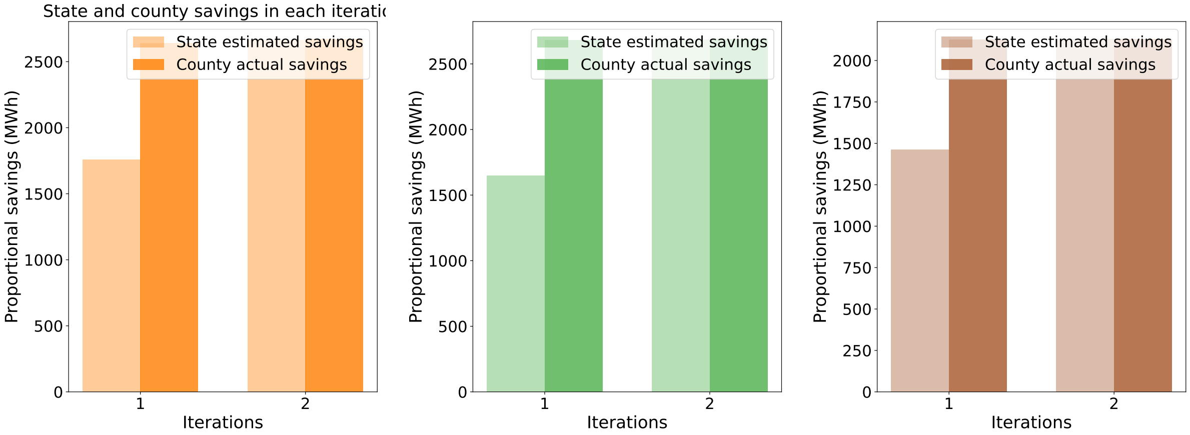

(a) Low I-Wϵ Index (b) Medium I-Wϵ Index (c) High I-Wϵ Index

Fig. 12: **State and county savings comparisons over iterations for different I-Wϵ Index settings.** Each panel shows the evolution of savings at both state and county levels across iterations under different I-Wϵ Index values.

We analyze the convergence of our iterative framework across three different I-Wϵ Index settings: low, medium, and high. The low setting is defined by an ϵ value of 0.5, the medium setting by an ϵ of 0.7, and the high setting by an ϵ of 0.9. For these experiments, we used a λ value of 0.99 to introduce a small feedback from the county-level experiments. The objective of the state-level approach for iteration 1 remains the same as the objective in Equation P1 in the main paper. Starting from iteration 2, the state-level objective is updated to $\max_y \sum_{k=1}^{M} \left[\lambda y_k r_k + (1-\lambda)\frac{S^{(t-1)}}{y_k^{(t-1)}}\right]$, where t denotes the current iteration number, y_k is the budget allocation to county k, r_k is the average savings rate, and $S^{(t-1)}$ represents the total savings from previous iteration, which is given by $S^{(t-1)} = \sum_{k=1}^{M} y_k^{(t-1)} r_k^{(t-1)}$. The county-level objective remains the same as Equation P2 in the main manuscript and takes the input from the output of the state-level problem at iteration t.

The iterative framework converged between the state and county-level savings as follows: round 2 for $\epsilon = 0.5$, round 2 for $= 0.7$, and round 2 for $\epsilon = 0.9$ as shown in Figure 12 The absolute relative percentage difference between the state and the county savings are 0.45%,

0.06%, and 0.024% for ϵ of 0.5, 0.7, and 0.7, respectively. Although the state's initial estimated savings are lower than the actual savings achieved by the county in the first round, the county's feedback on its budget allocation helps the state adjust its budget to better align with the county's actual savings.

Notation Table

We list the notations used in our formulations in Table 2.

**Variable**	**Description**
M	Number of counties
N	Number of households
r_i	Retrofitting cost of i household
s_i	Energy savings from i household
w_i	Weight of i household
W_k	Weight of kth county
$\mathcal{C}_k$	set of households in k^{th} county
ϵ	equity/distribution parameter
$\mathcal{B}$	Total allocated budget
x_i	Allocated budget for household i
y_k	Allocated budget for county k
r_k	Average savings rate
λ	Feedback parameter
$\mathsf{B}_{\text{state}}$	Lifted variable for the state
B_{k}	Lifted variable for the county k
$f(\epsilon)$	Scalar function of ϵ
$\mathcal{F}(x, w)$	IW$_\epsilon$ Index with weight w
$w_{\mathcal{C}_k}$	Weight for household in k^{th} county
$x_{\mathcal{C}_k}$	Allocated budget for household in k^{th} county
z_k	Auxiliary variable for the county k
z_i	Auxiliary variable for the household i
n_k	Number of households in the county k
t	Current iteration number

Table 2. Notation table for the variables used in this work.

Approach to solve the equity constraint

The equity constraint $\mathcal{F}(x, w) \geq \epsilon$ is as a second-order cone (SOC) at both the state and county levels. The SOC constraint, as presented in Equation (1) in the main manuscript, is a $(n+1)$ dimensional SOC constraint. Despite being convex, it is known in the literature that when solving a problem that includes a high dimensional SOC, for the sake of computational efficiency, it is always better to disaggregate this $(n + 1)$-dimensional SOC into n, three-dimensional rotated second-order cone (R-SOC) constraints and in turn outer-approximate the same [6]. The disaggregation procedure is performed by introducing additional auxiliary or lifted variables. We represent the disaggregated R-SOC constraints using lifted variables, as shown below.
State-level:
We introduce a lifted variable $\mathsf{B}_{\text{state}}$, defined as a scaled version of the ℓ_1-norm of the element-wise product $w \circ y$, defined as:

$$\sum_{k=1}^{M} W_k y_k = f(\epsilon) \cdot \mathsf{B}_{\text{state}} \qquad \text{(Lifted Variable – State)}$$

where W_k is the state-level weight for county k, and $f(\epsilon) = (1 - \epsilon) + \epsilon\sqrt{M}$ is a scalar scaling function.

To approximate the ℓ_2-norm of the weighted vector $W \circ y$, we introduce auxiliary variables z_k for each county k:

$$\sum_{k=1}^{M} z_k = \mathsf{B}_{\text{state}} \qquad \text{(Auxiliary Constraint – State)}$$

$$z_k \cdot \mathsf{B}_{\text{state}} \geq (W_k y_k)^2 \quad \forall k \in \{1, \dots, M\} \qquad \text{(R-SOC – State)}$$

County-Level (for county k):
We apply a similar approach within each county. We define a lifted variable B_k as a scaled version of the ℓ_1-norm of the vector $w_{\mathcal{C}_k} \circ x_{\mathcal{C}_k}$, using:

$$\sum_{i \in \mathcal{C}_k} w_i x_i = f(\epsilon) \cdot \mathsf{B}_k \qquad \text{(Lifted Variable – County } k\text{)}$$

where $f(\epsilon) = (1-\epsilon) + \epsilon\sqrt{n_k}$ and $n_k = |\mathcal{C}_k|$ is the number of households in county k.

We then introduce auxiliary variables z_i for each household $i \in \mathcal{C}_k$:

$$\sum_{i \in \mathcal{C}_k} z_i = \mathsf{B}_k \qquad \text{(Auxiliary Constraint – County } k\text{)}$$

$$z_i \cdot \mathsf{B}_k \geq (w_i x_i)^2 \quad \forall i \in \mathcal{C}_k \qquad \text{(R-SOC – County } k\text{)}$$

These constraints help to solve the equity metric $\mathcal{F}(x_{\mathcal{C}_k}, w_{\mathcal{C}_k}) \geq \epsilon$.

Comparison between alternate single-level and two-level formulations for retrofitting budget allocation

Table 3. Comparison of single-level and two-level optimization performance across counties.

County	Amount ($)	Population	Low $I\text{-}W\epsilon = 0.5$ Time (s)	Mid $I\text{-}W\epsilon = 0.7$ Time (s)	High $I\text{-}W\epsilon = 0.9$ Time (s)	Low $I\text{-}W\epsilon = 0.5$ Saving (kWh)	Mid $I\text{-}W\epsilon = 0.7$ Savings (kWh)	High $I\text{-}W\epsilon = 0.9$ Savings (kWh)
Single-level optimization								
Two	176M	485,923	4356	3508	3650	487,107.26	405,056.29	314,311.79
Three	216M	604,446	6276	4766	7512	601,930.26	499,869.33	386,994.53
Four	260M	727,597	6405	7554	7393	798,850.03	667,078.09	522,372.44
Five	292M	840,020	8243	7034	10923	959,878.44	797,676.01	624,818.10
Two-level optimization								
Two	176M	485,923	455	426	398	437,955.63	383,651.23	306,408.83
Three	216M	604,446	456	489	459	548,019.65	479,027.16	380,187.06
Four	260M	727,597	553	489	460	785,818.24	695,213.39	542,811.84
Five	292M	840,020	431	497	434	979,627.31	856,302.60	659,351.30

As mentioned at the end of Methods Section in the main manuscript, an alternate formulation for Problem 1 can be set up where the IW_ϵ index for household-level can be replaced by$\mathcal{F}(x_N, w_N) \geq \epsilon$, where N is the total households.

We compare and discuss the computational time and solution for this alternate approach with our two-level approach given in P1-P2 (in the main manuscript), in Table 3. For this computational study, we consider households from two to five counties, selected in decreasing order of population. We pre-process the population based on the selection criteria outlined in Section 5. The timing comparison clearly demonstrates a significant speed-up achieved through two-level optimization compared to the single-level approach. Across all counties and varying $I - W\epsilon$ Index values, the two-level optimization shows a speed-up ranging from $9\times$ to $25\times$ compared to the single-level method. The two-level optimization achieves energy savings that

vary between -10% and +5% compared to the single-level approach. Negative values indicate cases where the two-level method results in slightly lower savings, while positive values represent scenarios where the two-level method yields greater savings than the single-level. It is worth mentioning that the IW_ϵ Index constraints for households in the single and two-level formulations do not exactly overlap as the constraint depends not just on the value of ϵ but also on the size of population (see (1) in the main manuscript). For the two-level formulation, these constraints include households for each county, while in the single-level, it involves all households and hence has a much bigger value n. Given that the savings from either approach are roughly equivalent, changing the value of ϵ between the two approaches can minimize the difference even further. However, the two-level formulations improved computational performance, which makes it the ideal framework for implementation.

References

1. Ayman Alazazmeh and Muhammad Asif. Commercial building retrofitting: Assessment of improvements in energy performance and indoor air quality. *Case Studies in Thermal Engineering*, 26:100946, 2021.
2. Ehsan Asadi, Manuel Gameiro Da Silva, Carlos Henggeler Antunes, and Luís Dias. Multi-objective optimization for building retrofit strategies: A model and an application. *Energy and Buildings*, 44:81–87, 2012.
3. Fabrizio Ascione, Nicola Bianco, Rosa Francesca De Masi, Gerardo Maria Mauro, and Giuseppe Peter Vanoli. Energy retrofit of educational buildings: Transient energy simulations, model calibration and multi-objective optimization towards nearly zero-energy performance. *Energy and Buildings*, 144:303–319, 2017.
4. Fabrizio Ascione, Rosa Francesca De Masi, Filippo de Rossi, Silvia Ruggiero, and Giuseppe Peter Vanoli. Optimization of building envelope design for nzebs in mediterranean climate: Performance analysis of residential case study. *Applied energy*, 183:938–957, 2016.
5. Dominic J. Bednar, Tony G. Reames, and Gregory A. Keoleian. The intersection of energy and justice: Modeling the spatial, racial/ethnic and socioeconomic patterns of urban residential energy use and efficiency. *Energy and Buildings*, 143:95–106, 2017.
6. Abhay Singh Bhadoriya, Deepjyoti Deka, and Kaarthik Sundar. Equitable routing–rethinking the multiple traveling salesman problem. *arXiv preprint arXiv:2404.08157*, 2024.
7. Benigna Boza-Kiss, Sergi Moles-Grueso, and Diana Urge-Vorsatz. Evaluating policy instruments to foster energy efficiency for the sustainable transformation of buildings. *Current Opinion in Environmental Sustainability*, 5(2):163–176, 2013.
8. Harriet Bulkeley, Pauline M. McGuirk, and Gareth Powells. Transitioning from urban climate action to climate equity: Governance, justice and the politics of transition. *Energy Research & Social Science*, 79:102187, 2021.
9. Sanya Carley and David M. Konisky. A review of the environmental policy literature from 2014 to 2017 with a closer look at the energy justice field. *Policy Studies Journal*, 48(S1):S7–S40, 2020.
10. Wen Wei Che, Chi Yan Tso, Li Sun, Danny YK Ip, Harry Lee, Christopher YH Chao, and Alexis KH Lau. Energy consumption, indoor thermal comfort and air quality in a commercial office with retrofitted heat, ventilation and air conditioning (hvac) system. *Energy and Buildings*, 201:202–215, 2019.
11. Saleh Abu Dabous and Fatma Hosny. A review of building envelope retrofitting methods for improving energy efficiency, aesthetic, and indoor environmental quality. *Energy Nexus*,

page 100407, 2025.
12. Maria Ferrara, Enrico Fabrizio, Joseph Virgone, and Marco Filippi. A simulation-based optimization method for cost-optimal analysis of nearly zero energy buildings. *Energy and Buildings*, 84:442–457, 2014.
13. Benjamin Goldstein and et al. Racial inequity in household energy efficiency and carbon emissions. *One Earth*, 5:1061–1071, 2022.
14. Najme Hashempour, Roohollah Taherkhani, and Mahdi Mahdikhani. Energy performance optimization of existing buildings: A literature review. *Sustainable Cities and Society*, 54:101967, 2020.
15. Diana Hernández. Understanding "energy insecurity" and why it matters to health. *Social Science & Medicine*, 167:1–10, 2016.
16. Diana Hernández. Energy insecurity and its ill health effects: A community perspective. *Energy Research & Social Science*, 56:101–106, 2018.
17. Diana Hernández and Jessica Laird. Surviving a shut-off: U.s. households at greatest risk of utility disconnections and how they cope. *American Behavioral Scientist*, 2021.
18. Kirsten E. H. Jenkins, Jennie C. Stephens, and Tony G. Reames. Incorporating energy justice throughout clean-energy r&d in the united states: A review of outcomes and opportunities. *Energy Research & Social Science*, 80:102212, 2021.
19. Džana Kadrić, Amar Aganović, and Edin Kadrić. Multi-objective optimization of energy-efficient retrofitting strategies for single-family residential homes: Minimizing energy consumption, co2 emissions and retrofit costs. *Energy Reports*, 10:1968–1981, 2023.
20. DEKD Kolokotsa, D Rovas, E Kosmatopoulos, , and K Kalaitzakis. A roadmap towards intelligent net zero-and positive-energy buildings. *Solar energy*, 85(12):3067–3084, 2011.
21. J. et al. Lewis. Energy efficiency as energy justice: Addressing racial inequities. *American Journal of Public Health*, 109(12):e1–e2, 2019.
22. Guopeng Li, Yingqing Xu, and Yue Fan. The design of sustainable retrofitting strategies and energy-efficiency optimization for residential buildings. In *IOP Conference Series: Materials Science and Engineering*, volume 556, page 012054. IOP Publishing, 2019.
23. Jing Liang, Yueming Qiu, Timothy James, Benjamin L Ruddell, Michael Dalrymple, Stevan Earl, and Alex Castelazo. Do energy retrofits work? evidence from commercial and residential buildings in phoenix. *Journal of Environmental Economics and Management*, 92:726–743, 2018.
24. Cara H Lozinsky, Núria Casquero-Modrego, and Iain S Walker. The health and indoor environmental quality impacts of residential building envelope retrofits: A literature review. *Building and Environment*, page 112568, 2025.
25. Giacomo Luddeni, Moncef Krarti, Giovanni Pernigotto, and Andrea Gasparella. An analysis methodology for large-scale deep energy retrofits of existing building stocks: Case study of the italian office building. *Sustainable Cities and Society*, 41:296–311, 2018.
26. Rit Martin, Thomas Arthur, Villot Jonathan, Thorel Mathieu, Garreau Enora, and Girard Robin. Shape: A temporal optimization model for residential buildings retrofit to discuss policy objectives. *Applied Energy*, 361:122936, 2024.
27. Sobhi Mejjaouli and Maha Alzahrani. Decision-making model for optimum energy retrofitting strategies in residential buildings. *Sustainable Production and Consumption*, 24:211–218, 2020.
28. Silvia Perez-Bezos, Olatz Grijalba, and Olatz Irulegi. Proposal for prioritizing the retrofitting of residential buildings in energy poverty circumstances. *Rigas Tehniskas Universitates Zinatniskie Rakst*i, 24(3):66–79, 2020.

29. Tony G. Reames. Targeting energy justice: Exploring spatial, racial/ethnic and socioeconomic disparities in urban residential heating energy efficiency. *Energy Policy*, 97:549–558, 2016.
30. Sunil Kumar Sharma, Swati Mohapatra, Rakesh Chandmal Sharma, Sinem Alturjman, Chadi Altrjman, Leonardo Mostarda, and Thompson Stephan. Retrofitting existing buildings to improve energy performance. *Sustainability*, 14(2):666, 2022.
31. Lei Shu, Tianzhen Hong, Kaiyu Sun, and Dong Zhao. Framework to select robust energy retrofit measures for residential communities. *Energy and Buildings*, 327:115077, 2025.
32. Benjamin K. Sovacool, Matthew Burke, Lucy Baker, Chaitanya K. Kotikalapudi, and Holle L. Wlokas. Evaluating equity and justice in low-carbon energy transitions. *Nature Energy*, 4:42–47, 2019.
33. Swapna Thorve, Young Yun Baek, Samarth Swarup, Henning Mortveit, Achla Marathe, Anil Vullikanti, and Madhav Marathe. High resolution synthetic residential energy use profiles for the united states. *Scientific Data*, 10(1):76, 2023.
34. U.S. Department of Energy. Methodology for evaluating cost-effectiveness of commercial energy code changes. `https://www.energycodes.gov/sites/default/files/2021-07/commercial_methodology.pdf`, July 2021. Federal Energy Management Program.
35. Griet Verbeeck and Hugo Hens. Energy savings in retrofitted dwellings: economically viable? *Energy and buildings*, 37(7):747–754, 2005.
36. Virginia Department of Energy. Energy efficiency and conservation block grant (eecbg) subgrants. `https://energy.virginia.gov/energy-efficiency/eecbg-subgrants.shtml`, 2024. Accessed May 27, 2025.
37. Nicola Willand, Trivess Moore, Ralph Horne, and Sarah Robertson. Retrofit poverty: Socioeconomic spatial disparities in retrofit subsidies uptake. *Buildings & Cities*, 1(1), 2020.
38. Haonan Zhang, Kasun Hewage, Hirushie Karunathilake, Haibo Feng, and Rehan Sadiq. Research on policy strategies for implementing energy retrofits in the residential buildings. *Journal of Building Engineering*, 43:103161, 2021.
39. Zhihua Zhou, Shuzhen Zhang, Chendong Wang, Jian Zuo, Qing He, and Raufdeen Rameezdeen. Achieving energy efficient buildings via retrofitting of existing buildings: a case study. *Journal of Cleaner Production*, 112:3605–3615, 2016.
